# Deep learning–enabled scaffolding of spatial arrays of PfCSP epitopes

**DOI:** 10.1073/pnas.2521914123

**Published:** 2026-04-07

**Authors:** Nelson R. Wu, Karla M. Castro, Nathan Beutler, Wen-Hsin Lee, Sai S. R. Raghavan, Gregory M. Martin, Monika Jain, Sashank Agrawal, Alessia Liguori, Oleksandr Kalyuzhniy, Patrick D. Skog, Sierra Terada, Yen-Chung Lai, Justin Ndihokubwayo, Danny Lu, Saman Eskandarzadeh, Nushin Alavi, Nicole Phelps, Ryan Tingle, John E. Youhanna, Sonya Amirzehni, Thomas F. Rogers, Dennis R. Burton, Ian A. Wilson, Andrew B. Ward, Bruno E. Correia, William R. Schief

**Affiliations:** ^a^Department of Immunology and Microbiology, The Scripps Research Institute, La Jolla, CA 92037; ^b^Institute of Bioengineering, École Polytechnique Fédérale de Lausanne, Lausanne 1015, Switzerland; ^c^Department of Integrative Structural and Computational Biology, The Scripps Research Institute, La Jolla, CA 92037; ^d^IAVI Neutralizing Antibody Center, The Scripps Research Institute, La Jolla, CA 92037; ^e^Division of Infectious Diseases, Department of Medicine, University of California, San Diego, CA 92037; ^f^The Ragon Institute of Mass General Brigham, Massachusetts Institute of Technology and Harvard University, Cambridge, MA 02139; ^g^The Skaggs Institute for Chemical Biology, The Scripps Research Institute, La Jolla, CA 92037; ^h^Moderna, Inc., Cambridge, MA 02139

**Keywords:** malaria, deep learning, epitope scaffold, vaccine, protein structure

## Abstract

Malaria is a leading cause of disease in developing countries. The licensed malaria vaccine RTS,S/AS01 confers partial protection in part due to the elicitation of circumsporozoite protein (CSP) antibodies, of which those to the CSP repeat and junctional regions offer the most potent protection. Anti-repeat region antibodies, including the protective antibody L9, frequently develop mutations that promote inter-Fab contacts when bound to CSP in “spiral” quaternary structures. As a first step toward the design of immunogens that elicit L9-like antibodies, we utilized generative deep learning models to design epitope scaffolds that incorporated up to three junctional repeat epitopes with structural conformations and relative spatial orientations matching those of the multivalent complex of CSP bound to three copies of L9. Affinity and structural studies demonstrated accurate scaffolding of two epitopes with the intended relative orientation, and displacement of the third epitope, while maintaining inter-Fab contacts between L9 antibodies. In a mouse model of malaria liver invasion, immunization with nanoparticles displaying these scaffold immunogens inhibited liver invasion as potently as matched nanoparticles displaying a short junctional peptide but less potently than the same nanoparticles displaying longer junctional peptides. This study demonstrates a substantial advance for design of multiepitope scaffolds with predetermined relative epitope spatial positioning. The study also represents an initial step toward development of multiepitope immunogens to elicit antibodies that utilize homotypic interactions to bind pathogens in multivalent clusters.

## Significance

Malaria is a leading cause of disease in developing countries. Monoclonal antibody L9 has been shown to provide potent protection against malaria in humans. L9 binds to NPNV motifs within the circumsporozoite protein (CSP), with three L9 Fabs each interacting with one NPNV motif and an adjacent Fab. We utilized generative deep learning models to design epitope scaffolds that incorporated up to three NPNV motifs with structural conformations and relative spatial orientations matching those of the multivalent complex of CSP bound to three copies of L9. Affinity and structural studies demonstrated accurate scaffolding in two of three epitopes in the most complex scaffold. This study demonstrates a substantial advance for design of multiepitope scaffolds with predetermined relative epitope spatial positioning.

Malaria is a tropical disease caused by *Plasmodium* parasites of which *falciparum* is the cause of most malaria-related mortality (1, 2). *Plasmodium falciparum* sporozoites have a surface coating of circumsporozoite protein (PfCSP), which comprises an amino-terminal domain, a junctional domain consisting of repeats of DPNA and NPNV sequence motifs, a central region of highly conserved NANP and NANP-like motifs repeated approximately 37 to 70 times depending on the strain, and a carboxyl-terminal domain. Leading vaccines, RTS,S and R21, display 19 NANP major repeats and the C-terminal region on a virus-like particle with hepatitis B surface antigen (3). Clinical trials with the RTS,S/AS01 vaccine showed variable levels of efficacy that waned over time (3). R21 was found to reach up to 75% efficacy against infection in a subset of African children, surpassing the efficacy of RTS,S, but the durability of protection remains to be determined (4).

Monoclonal antibody (mAb) L9 is one of the most potently protective anti-malaria mAbs in preclinical models (5) and in humans (6, 7). L9 binds specifically and multivalently to NPNV motifs within the junctional region (5, 8). Cryoelectron microscopy (cryoEM) studies show that three L9 Fabs bind to one recombinant CSP molecule, with each Fab interacting with one NPNV motif and with an adjacent Fab through homotypic interactions, and the entire assembly forms a pseudospiral (9, 10). Somatic hypermutations can occur at these inter-Fab interfaces and, when present, inter-Fab contacts are associated with improved protection (11). Three NPNV motifs separated by two tetrapeptide motifs, as in the PfCSP constructs characterized by cryoEM, are found in 83% of PfCSP isolates. Hence, the observed multivalent pseudospiral interaction could be relevant for broad malaria inhibition by L9 (9, 10). Furthermore, immunogens that reproduce the pseudospiral spatial arrangement of NPNV motifs in the L9-bound conformation have potential to elicit antibodies similar to L9, with similar NPNV specificity and homotypic interactions. Given the high potency of L9, this approach has potential to contribute to next-generation vaccines with improved efficacy.

The design of epitope scaffolds, where an antibody epitope is embedded into a scaffold protein that exposes the epitope and stabilizes a relevant (usually antibody-bound) conformation, has provided fertile ground for testing computational methods to manipulate protein structure and for exploring linkages between immunogen structure and immune responses. Early efforts focused on epitope grafting, either side-chain grafting (12–14) or backbone grafting (15, 16), to structurally suitable host scaffold proteins. Such epitope scaffolds were developed for both continuous (12–14, 16) and discontinuous epitopes (15) and were employed to engineer additional contacts to antibody with fixed spatial geometry relative to the main epitope (17), but the range of host scaffolds was limited to proteins of known structure. Furthermore, although such epitope scaffolds were shown to induce structure-specific antibodies in several studies (12–14), elicitation of neutralizing antibodies was not reported, which in some cases was attributed to a failure to scaffold complete antibody epitopes (18). Design of de novo scaffolds to fold around and stabilize a given epitope increased the flexibility and applicability of the epitope scaffolding approach, and de novo epitope scaffolds presenting neutralizing antibody epitopes from respiratory syncytial virus (RSV) were the first epitope scaffolds reported to elicit virus neutralizing antibodies (19, 20). Deep learning methods applied to motif grafting further expanded the range of accessible scaffold folds (21, 22) and have recently allowed design of single scaffolds presenting multiple different epitopes but in random relative positions (23). Here, we utilized deep learning generative models to design scaffolds hosting up to three NPNV repeat motifs with structural conformations and relative spatial orientations matching those of the multivalent complex of CSP bound to three copies of the highly protective antibody L9. We describe the design as well as the biophysical and structural evaluation of scaffolds with one, two, or three NPNV epitopes.

## Results

### Scaffolding NPNV Epitopes in Bound Conformation.

**Fig. 1.**
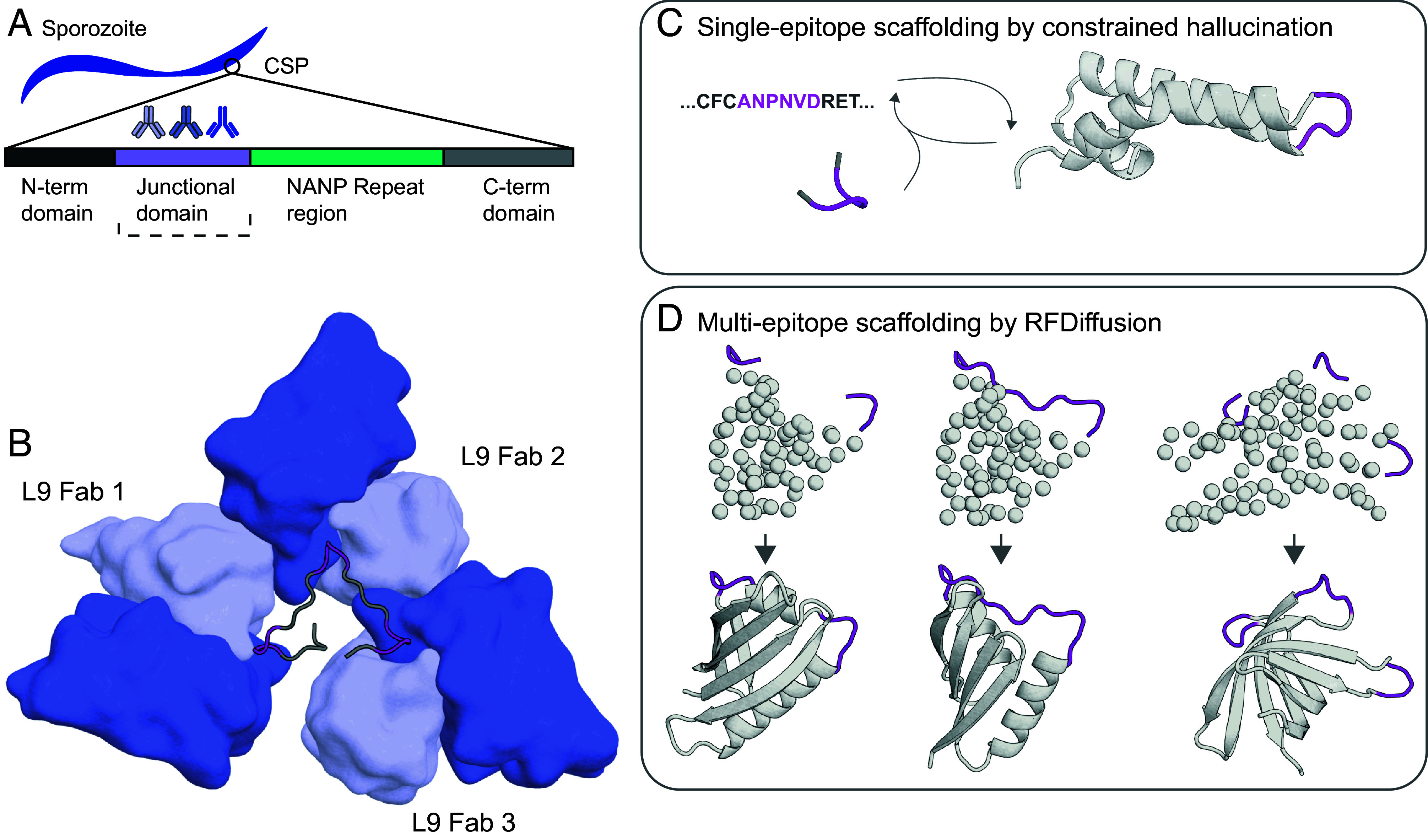
Computational scaffolding of NPNV immunogens. (*A*) The NANP repeat domain (green) and L9 targeted junctional region (purple) are represented on CSP on the parasite surface (not to scale). (*B*) Three L9 Fabs bound to NPNV epitopes. The CSP junctional region peptide is shown as a cartoon loop with the three NPNV epitopes highlighted in purple. The antibody variable domains are shown as surface representations with heavy and light chains represented in dark and light blue, respectively (PDB: 8EH5). (*C*) Constrained hallucination by RoseTTAFold updating sequence and structure in iterative cycles generated single-epitope scaffolds hosting one NPNV epitope in the L9-bound conformation. (*D*) RFdiffusion-generated scaffolds hosting multiple NPNV epitopes in L9-bound conformations and native relative orientations supporting a spiral structure through inter-Fab contacts.

Previously reported cryoEM structures of L9 Fab bound to a recombinant PfCSP construct revealed a complex with three L9 Fabs bound to one CSP peptide, with each Fab interacting with a junctional region NPNV motif and an adjacent Fab through homotypic interactions (9, 10) (Fig. 1 *A* and *B*). Although the NPNV motif is likely disordered in solution (24), each of the three L9-bound NPNV peptides was shown to adopt a similar loop structure, with RMSD values of 0.05 to 0.10 Å between the three NPNV epitopes (9, 10).

We hypothesized that deep learning could be used to scaffold natively disordered NPNV repeat epitopes in defined bound conformations and specifically, diffusion models would be able to generate viable designs for increased complexity of multiepitope scaffolding and native orientation presentation (Fig. 1 *C* and *D*).

**Fig. 2.**
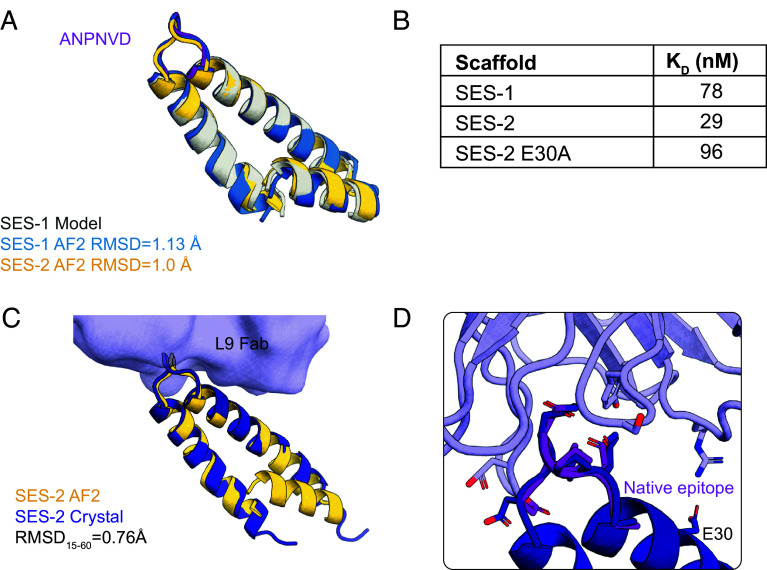
Characterization of single-NPNV epitope scaffolds. (*A*) RoseTTAFold models (gray) with highlighted NPNV epitope (purple) of SES-1 prior to ProteinMPNN redesign from yeast display overlaid on SES-1 AF2 prediction (blue) and further designed SES-2 AF2 prediction (yellow). (*B*) SPR affinity measurements of L9 Fab and L9 scaffolds. (*C*) Crystal structure of SES-2 bound to L9 Fab overlaid with the AF2 model showing close backbone agreement (RMSD = 0.76 Å) (26). (*D*) Superimposition of the native NPNV epitope in the bound conformation (PDB: 7QQR) on the grafted NPNV epitope with RMSD all atom of 1.34 Å. Additional contact residue E30 is labeled.

We first evaluated the ability of RoseTTAfold (8) to design single epitope scaffolds (SES), with one flanking residue at the N- and C terminus (ANPNVD), in the L9-bound conformation (PDB:7RQQ) (Fig. 1*C*). Ten ProteinMPNN (25) sequences for each of 19 selected backbone scaffolds were screened by three rounds of yeast surface display with increasingly stringent proteolytic pressure to eliminate unfolded sequences. When we determined the amino acid sequence for the highest affinity scaffold to L9, we noted that the scaffold contained many aliphatic surface residues. Upon producing recombinant protein, we observed precipitation at higher concentrations, which precluded structural determination by crystallography. This scaffold, termed SES-1, was further subjected to surface redesign to improve surface hydrophilicity, producing the scaffold SES-2 with positive surface charge. The AF2 model for SES-2 retained high fidelity to the starting backbone (RMSD_backbone_ = 1.0 Å) (Fig. 2*A*). The SES-1 and SES-2 scaffolds showed alpha-helical profiles by circular dichroism (CD) (*SI Appendix*, Fig. S1) and had melting temperatures from differential scanning calorimetry (DSC) of 60 °C and 54 °C, respectively (*SI Appendix*, Fig. S2). SES-1 was shown by SEC-MALS to be a soluble monomer, and SES-2 was found to be a soluble dimer after redesign (*SI Appendix*, Fig. S14). Scaffold interactions with L9 by surface plasmon resonance (SPR) were done with scaffold captured on the sensor and L9 Fab flowed as analyte to account for avidity; SES-1 had a dissociation constant [K_D_] of 78 nM, whereas SES-2 had modestly higher affinity (K_D_, 29 nM) (Fig. 2*B* and *SI Appendix*, Fig. S12). Previously, the dissociation constant of L9 to a flexible single-NPNV repeat peptide was reported as 1,900 nM (8), a value that is 24- to 65-fold lower than the affinities we measured for L9 to SES-1 and SES-2, respectively.

To assess the conformation of the grafted NPNV epitope, we determined the structure of SES-2 bound to L9 Fab by x-ray crystallography (26). We observed good agreement between the overall structure of the coiled-coil segment of SES-2 in the crystal structure and the predicted structure by AF2 (Residues 15 to 60 RMSD_backbone_ = 0.76 Å) (Fig. 2*C*). However, the first N-terminal helix was unresolved in the crystal structure, likely due to flexibility. The scaffolded NPNV epitope showed high structural similarity to the native epitope structure (RMSD_all-atom_= 1.34 Å) (Fig. 2*D*) (PDB: 7QQR). We found one additional, unintentional salt bridge between L9 light chain residue R31 and the scaffold residue E30; by mutating this glutamic acid to alanine, we found that this interaction accounted for the small affinity increase from SES-1 to SES-2 (Fig. 2 *B* and *D* and *SI Appendix*, Fig. S12). Taken together, these results supported the use of deep learning to design scaffolds for single NPNV epitopes in the L9-bound epitope conformation with high affinity and structural fidelity.

### Scaffolding Multiple NPNV Epitopes with Native Orientation.

We next explored whether multiple NPNV epitopes could be scaffolded to present the L9-bound conformation in predetermined relative positions resembling the three L9-complexed NPNV repeats in the structure of recombinant CSP bound to L9 Fabs. We utilized RFdiffusion (22) to graft two and three discontinuous NPNV epitopes (PDB:8EH5). Aiming to produce at least 1,000 scaffold backbones for each design strategy, we designed 1,170 unique backbones where two NPNV repeats were grafted discontinuously and 1,257 unique backbones where the native linker residues between two NPNV repeats were grafted as well (Fig. 1*D*); 1,287 unique backbones were also generated by scaffolding all three epitopes discontinuously in three epitope orders within the linear scaffold sequence. By labeling NPNV epitopes in order as they appear in the native CSP sequence as A, B, and C, order-1 was defined as ABC, order-2 was defined as ACB, and order-3 was defined as CAB; diffusion using the other possible orders of CBA, BAC, and BCA failed to generate backbones with secondary structure. After filtering for globular structures, 7 double-NPNV backbones with native linkers, 6 double-NPNV backbones with de novo designed linkers, and 10 triple-NPNV backbones were selected for sequence design by ProteinMPNN. After designing multiple sequences for each backbone with ProteinMPNN, 8,515 double-NPNV scaffold designs and 4,411 triple-NPNV scaffold designs were selected by Alphafold2 (27) metrics for pLDDT model confidence and the TM-score (28) measure of similarity between designed structure and predicted structure. Designs with TMscore > 0.8 were ranked by highest pLDDT and lowest PAE. The top 64 double-NPNV designs and top 64 triple-NPNV designs by these metrics were screened separately using yeast display with increasingly stringent proteolytic pressure to eliminate unfolded sequences.

**Fig. 3.**
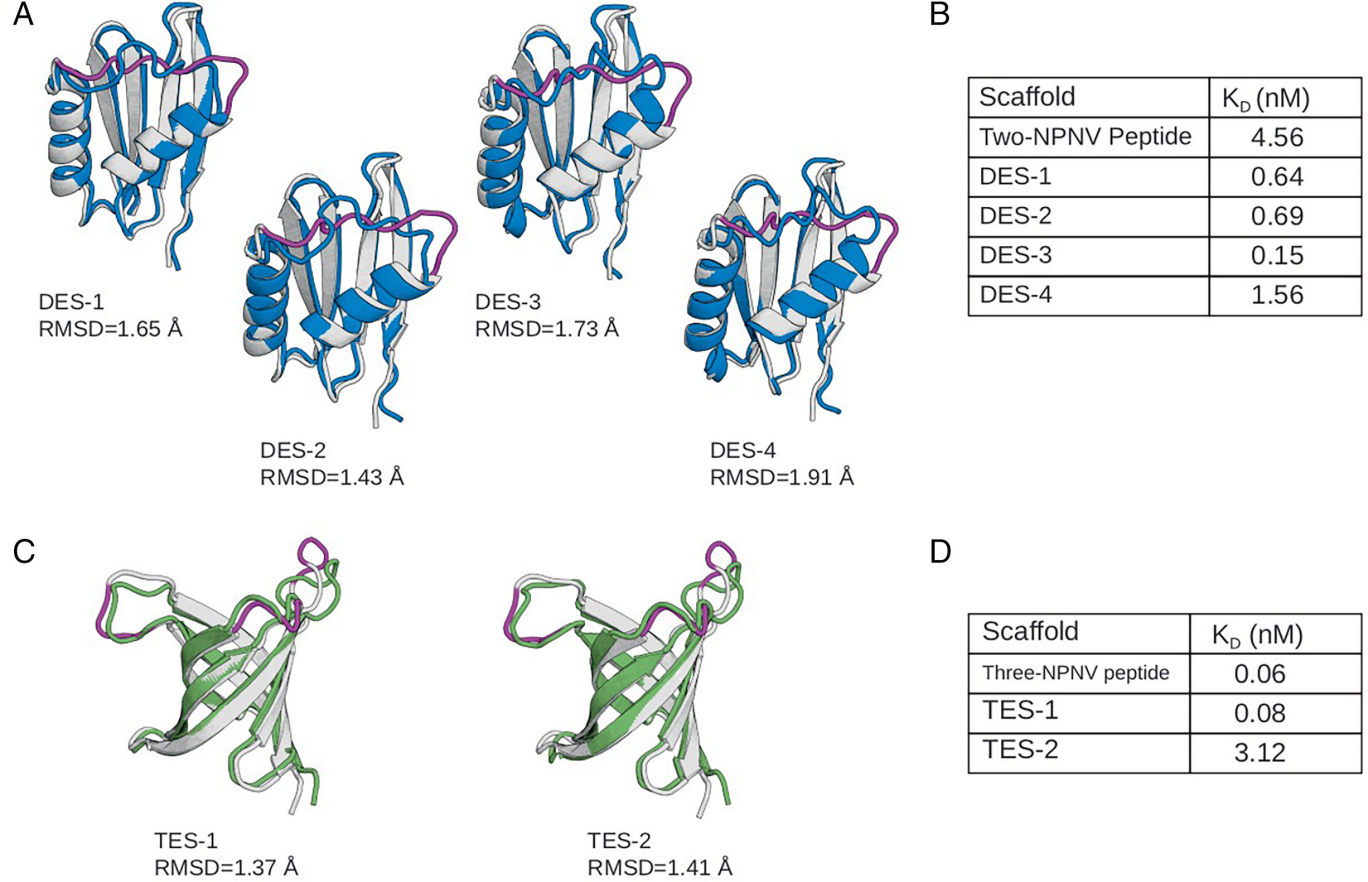
Characterization of multi-NPNV epitope scaffolds. (*A*) DES RFdiffusion models (gray) with repeat-linker region (purple) overlaid on the MPNN AF2 model (green). (*B*) Kinexa measurements for DES designs compared to a flexible double-NPNV peptide. (*C*) TES RFdiffusion models (gray) with repeat-linker region (purple) overlaid on the ProteinMPNN AF2 model (green). (*D*) Kinexa measurements for TES designs compared to a flexible triple-NPNV peptide.

From the yeast screen, we converged on and isolated four double-NPNV de novo linker designs, referred to as double-epitope scaffolds (DES). These scaffolds, termed DES-1, DES-2, DES-3, and DES-4, displayed mixed alpha-helical and beta-sheet secondary structure characteristics in CD experiments (*SI Appendix*, Fig. S1), as expected from the design models, and had melting temperatures from DSC of 92 °C, 99 °C, 56 °C, and 55 °C, respectively (*SI Appendix*, Fig. S2). To recapitulate the effect of inter-Fab contacts between L9 Fabs, we measured affinities using KinExA, which directly measures the concentration of free binding partner in solution after binding equilibrium has been reached. In the experimental design that we employed, KinExA would report monovalent dissociation constants. We found that three scaffolds had higher L9 affinity than the double-NPNV peptide (K_D_, 4.56 nM), with scaffold K_D_ values ranging from 151 pM (30-fold higher affinity) to 694 pM (6.6-fold higher affinity) (Fig. 3 *A* and *B*).

Two triple-NPNV scaffolds (triple-epitope scaffolds, TES) were isolated from yeast display screening, and both were from grafting order-3. Both were designed to be beta sheet proteins. Circular dichroism showed beta sheet profiles (*SI Appendix*, Fig. S1), and DSC showed high melting temperatures of 73 °C and 91 °C for TES-1 and TES-2, respectively (*SI Appendix*, Fig. S2). Using KinExA, we found that neither of the tested TES designs had higher L9 affinity than a triple-NPNV peptide (K_D_, 59 pM), although TES-1 had similar affinity to the triple-NPNV peptide (K_D_, 85 pM) and that same scaffold had 54-fold higher affinity compared to a double-NPNV peptide (K_D_, 4.56 nM) (Fig. 3 *C* and *D*). The fact that none of our order-1 or order-2 TES passed the yeast display screen demonstrated that the order of the repeats was an important factor in the success or failure of the computational design processes.

### Structurally Accurate Design of Multi-NPNV Scaffolds.

**Fig. 4.**
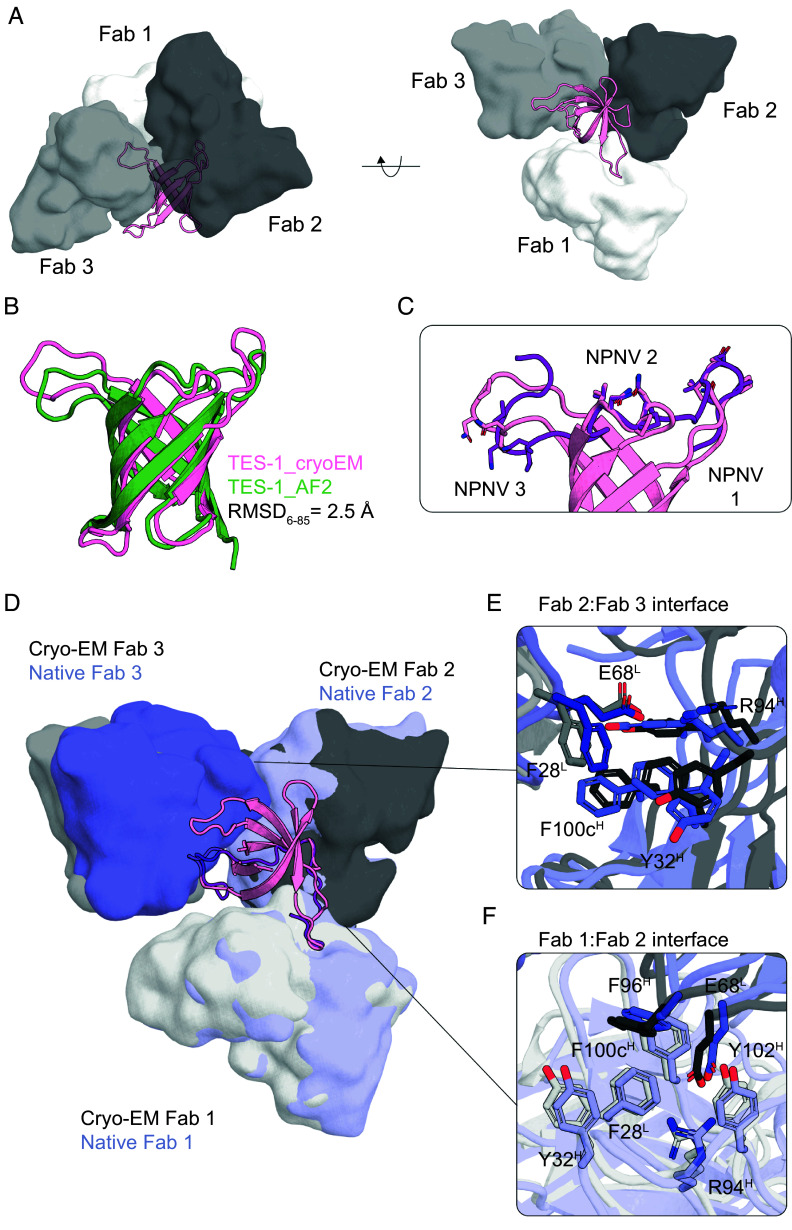
L9-bound multi-NPNV epitope scaffolds recapitulate inter-Fab contacts. (*A*) CryoEM structure of TES-1 (pink) in complex with three L9 Fabs (gray) (29, 30). (*B*) TES-1 cryoEM structure (pink) overlaid with the AF2 model (green) (29). (*C*) The *Left* panel shows the native structure of CSP-junctional repeats (purple) in complex with three L9 Fabs (blue) in bound orientation (PDB: 8EH5). The *Right* panel shows the native NPNV 1 and NPNV 2 epitopes (purple) with their bound Fab in native orientation overlaid with the cryoEM structure of TES-1 (pink) and three bound L9 Fabs (gray). Fabs 1 and 2 align with native orientations and Fab 3 hinges outward. (*D*) Superimposition of the three native NVNP epitopes in the bound conformation and relative orientation (PDB: 8EH5) on the grafted NVNP epitope. (*E* and *F*) CryoEM (gray) inter-Fab contacts between Fab2:Fab3 and Fab1:Fab2, respectively, overlaid on native Fab contacts (blue).

To examine the design accuracy of the repeat scaffolds and to further evaluate the affinity improvements observed in vitro, we determined the structure of the complex of TES-1 with L9 Fab by cryoelectron microscopy (29, 30). We found that all three NPNV motifs were occupied by L9 Fabs and that the three L9 Fabs made inter-Fab contacts similar to those in the multivalent complex of L9 Fabs with CSP peptide (Fig. 4*A*). The scaffold structure had a backbone RMSD for residues 6 to 85 resolved in the cryoEM structure of 2.50 Å compared to the AF2 structure model (Fig. 4*B*). Disregarding the epitope-hosting loops that are difficult for AF2 to predict, the beta-barrel structure of the protein was accurately modeled (backbone RMSD 1.94 Å). Each epitope aligned well to the corresponding epitope in the L9-bound peptide structure (all-atom RMSD values of 0.61 to 0.99 Å). NPNV motifs 1 and 2 were accurately scaffolded in their intended relative positions, whereas NPNV 3 was offset from the intended position relative to NPNV 1 and 2 by a C_α_ RMSD distance of 6.3 Å across the NPNV residues (Fig. 4*C*). NPNV 3 was observed to be positioned within a long and largely flexible loop (Fig. 4*D*), longer and likely more flexible than the loops hosting the NPNV 1 and 2 motifs, which provided potential explanations for the discrepancy in positioning of the NPNV 3 motif and lack of affinity improvement over the triple-NPNV peptide (Fig. 3*D*).

In the TES-1/L9 complex, the inter-Fab contacts Fab1:Fab2 and Fab2:Fab3 were accurately recapitulated compared to contacts in the native peptide-L9 complex structure (8–10, 29, 30) (PDB: 8EH5) despite a Fab2:Fab3 offset (Fig. 4 *D–F*). The Fab1 light chain residues F28, and Fab2 heavy chain residues Y32, F96, and F100c responsible for a number of pi–pi stacking interactions were accurately represented in the Fab1:Fab2 interfaces and shifted in the Fab2:Fab3 interface of the TES-1/L9 complex. The hydrogen bond between light chain residue H70 and heavy chain residue Q1 was not recapitulated, likely as a result of the flexibility of the N terminus where Q1 is located. However, the CDRH3-stabilizing interaction critical for antigen interaction between heavy chain R94 and heavy chain Y102, and additional contact to light chain E68 was well recapitulated in the Fab1:Fab2 interface.

Overall, the Fab1:Fab2 interface was well recapitulated, and the Fab2:Fab3 interface, while incorporating the same interactions, was shifted in accordance with the NPNV 3 orientation. Taken together, these data suggested that deep learning approaches can scaffold more than one disordered repeat in its bound conformation and strikingly at least two epitopes in predetermined orientations, accurately recapitulating the inter-Fab contacts observed in native L9.

### Epitope Scaffolds Elicit Variable Levels of Inhibition in a Mouse Liver Invasion Model.

**Fig. 5.**
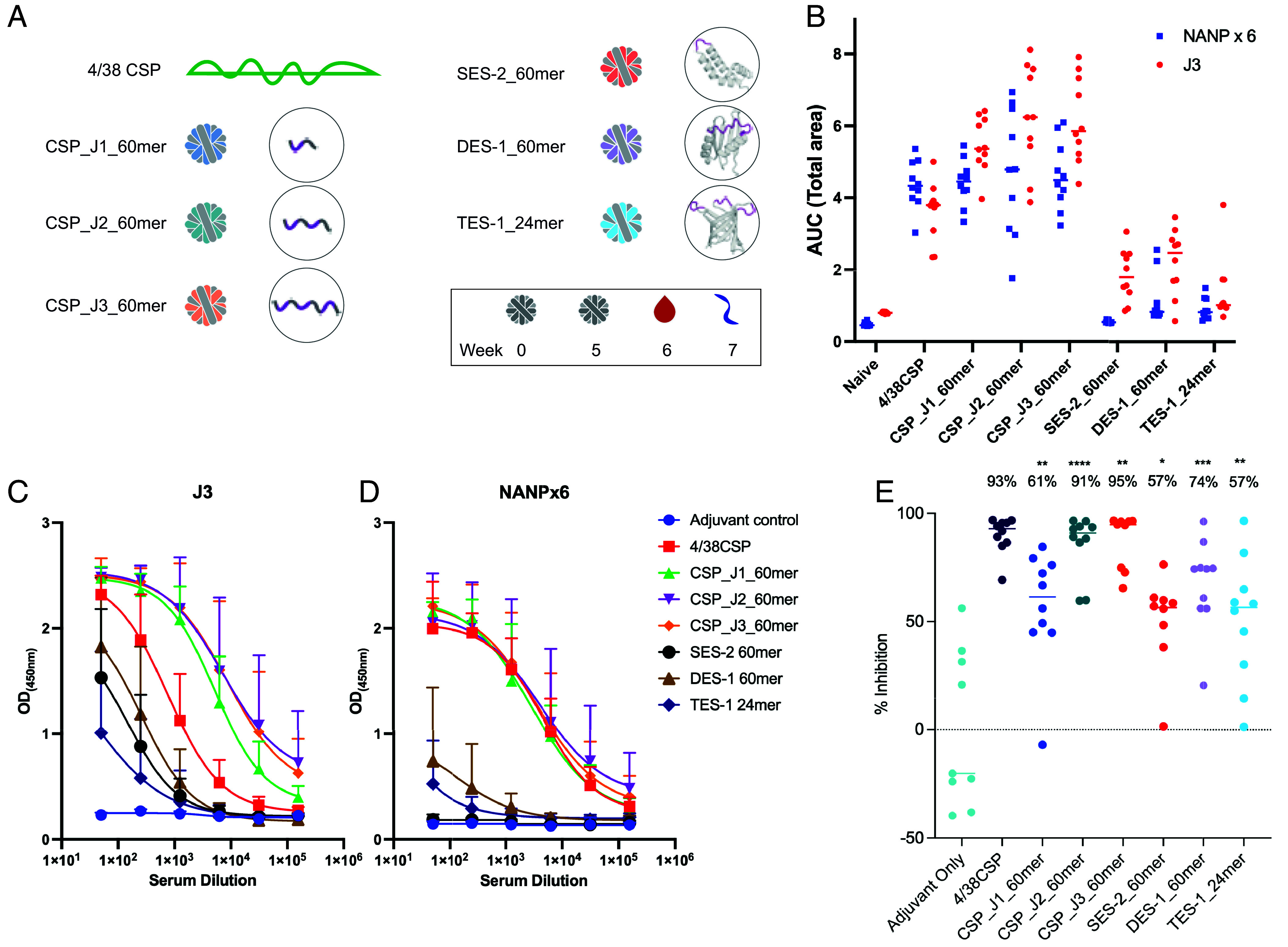
In vivo protection by de novo immunogens. (*A*) Representative diagrams of each nanoparticle with their corresponding CSP antigens. For peptide immunogens, CSP region of interest shown in purple. For scaffold immunogens, ribbon structures are shown with the CSP region of interest shown in purple. Mice were immunized at weeks 0 and 5, bled on week 6, and challenged on week 7. (*B*) Relative NANPx6 and J3 reactive serum IgG titers. ELISA plates were coated with a peptide composed of six sequential NANP repeats (NANPx6) or a peptide composed of three sequential junctional motifs (J3) (33) and incubated with sera. Area under the curve was calculated for each animal and antigen. Serum IgG binding curves to (*C*) J3 and (*D*) NANPx6 determined through ELISA. Each point represents the average OD for 9 to 10 mice in each immunogen group. (*E*) Mice (n = 10) were challenged with transgenic *Plasmodium berghei* expressing luciferase and *P. falciparum* CSP. After 42 h, mice were injected with D-luciferin and imaged. Flux values were normalized, and data were presented as % inhibition of liver invasion. Significance was established for all immunization groups. For comparisons to the adjuvant-only group, significance testing was performed using a two-tailed Mann–Whitney U test.

Given the initial scaffolding accuracy of the repeat epitopes, we evaluated in mice the immune responses and capacity to inhibit sporozoite invasion for selected scaffolds displayed on glycosylated lumazine synthase (60mer) or ferritin (24mer) nanoparticles. To evaluate the immunogenicity, we vaccinated C57BL/6J mice twice (week 0 and week 5) with proteins delivered with SMNP adjuvant (31). We tested the highest affinity single- and double-NPNV epitope scaffolds incorporated on 60mer nanoparticles (SES-2_60mer and DES-1_60mer), and the highest affinity triple-NPNV epitope scaffold on a 24mer nanoparticle (TES-1_24mer), as TES-1 failed to express on 60mer nanoparticles. As a positive control immunogen, we used full-length monomeric *Pf*CSP which contains four NPNV epitopes and 38 NANP epitopes (CSP 4/38) (32). Previously characterized 60mer nanoparticles displaying peptides encompassing the *Pf*CSP junctional region beginning with portion of the CSP N terminus (KLKQPADGNPDP) followed by either NANPNVDP (CSP_J1_60mer), NANPNVDPNANPNVDP (CSP_J2_60mer), or NANPNVDPNANPNVDPNANPNVDP (CSP_J3_60mer) were used as control comparisons (33) (Fig. 5*A* and *SI Appendix*, Table S3). Following a homologous boosting scheme (Fig. 5*A*), we evaluated antibody binding responses by ELISA using sera collected 1 wk after the second vaccination to a junctional peptide (J3) and major repeat peptide (NANPx6) (Fig. 5*C* and *SI Appendix*, Fig. S13). Antibody responses to both J3 and NANPx6 peptides were detected after vaccination with CSP4/38, CSP_J1_60mer, CSP_J2_60mer, and CSP_J3_60mer (Fig. 5 *B*–*D* and *SI Appendix*, Fig. S13). CSP4/38 contains both J3 and NANPx6 peptides within its sequence, and CSP4/38 vaccinated animals elicited antibody responses to both almost equally. J1, J2, and J3 peptides are included within the J3 peptide and contain no NANPx6 epitopes, and animals immunized with these junctional peptides elicited higher responses to the J3 peptide than NANPx6 peptide (Fig. 5 *B*–*D* and *SI Appendix*, Fig. S13). Reactivity to the J3 peptide elicited by scaffold nanoparticles was generally lower than that elicited by the J1, J2, or J3 peptide nanoparticles (Fig. 5 *B* and *C*), likely due at least in part to the greater number of epitopes and peptide conformations shared between the J1, J2, and J3 peptide immunogens and the J3 peptide ELISA antigen. The scaffold nanoparticles SES-2_60mer, DES-1_60mer, and TES-1_24mer elicited junctional-specific antibody responses with low specificity to the NANP major repeat peptide, similar to the binding profile observed for the L9 antibody (Fig. 5 *B*–*D* and *SI Appendix*, Fig. S13), and as expected since the scaffolds do not include the NANP major repeat peptide.

To measure the inhibition of sporozoite invasion provided by these immunogens, we performed retro-orbital sporozoite challenges at 2 wk postboost and monitored liver burden 42 h postchallenge as described previously (34). All results were normalized to the adjuvant-only control group in each study, and inhibition of liver invasion was calculated by reduction in liver burden signal compared to the adjuvant only group. Statistical significance in inhibition was determined using the Mann–Whitney test and noted when possible. All scaffolded immunogens and control immunogens elicited statistically significant levels of inhibition compared to the adjuvant only group (Fig. 5*E*) and higher levels of inhibition than nanoparticles containing no malaria epitopes previously reported for a separate experiment (33). Overall, the scaffolded immunogens provided lower levels of inhibition compared to the full-length CSP monomer control or nanoparticles displaying a portion of the CSP N terminus and at least two NPNV and two NANP epitopes (Fig. 5*E*). This was not entirely unexpected, given that the more epitope-rich peptide immunogens elicited higher circulating antibody titers than the scaffold immunogens to peptides J3 and NANPx6 that are found in the CSP produced by the challenge sporozoite specimens (Fig. 5 *B*–*D*). All peptide groups showed fold increases in titers against both peptides J3 and (NANPX6) compared to the scaffold groups, most likely resulting in higher levels of inhibition. Increasing the peptide length from J1 to J2 (*P* = 0.0015) or J3 (*P* = 0.0288) led to statistically significant increases in inhibition, although no significant difference was observed between J2 and J3 (Fig. 5*D*). In contrast, increasing the NPNV epitopes presented on the scaffold immunogens had no significant impact on J3 specific antibody titers or inhibition of liver invasion (Fig. 5 *B* and *E*). These data suggest that scaffolding natively disordered peptides conferred measurable but limited levels of inhibition.

## Discussion

The design of scaffold proteins that conformationally stabilize immunogenic epitopes or functional sites offers a wide range of potential applications in vaccine, therapeutic, and enzyme design. In this study, we evaluated the ability of generative learning–based design methods coupled with in vitro screening to design epitope scaffolds bearing multiple copies of a protective malaria antibody epitope in predefined relative orientations. Our investigation was motivated by the fact that the antibody L9 is highly protective against malaria in humans (6, 7) and the previous observation in cryoEM studies that interaction of L9 with CSP involves three L9 Fabs interacting with one CSP molecule in which each Fab interacts with one NPNV motif and also with an adjacent Fab through homotypic interactions (9, 10). We designed de novo epitope scaffolds attempting to present one, two, or three NPNV motifs, each conformationally stabilized in the L9-bound conformation and, in the cases of two or three NPNV motifs, the NPNV motifs were intended to be spatially arranged in relative positions as in the L9 interaction with recombinant CSP. Our biophysical and structural analyses indicated that the designs were relatively successful with, in particular, the cryoEM structure of a complex of a TES with three L9 Fabs showing that inter-Fab contacts were recapitulated in both Fab1:Fab2 and Fab2:Fab3 interfaces. Although the conformations of each of the NPNV motifs accorded well with the template L9-CSP structure, and the positions and orientations of NPNV 1 and NPNV 2 were essentially as intended, the placement of NPNV 3 was offset by a modest degree from the intended position. The location of NPNV 3 on an extended loop likely contributed to the reduced accuracy of its placement and may help explain the lack of affinity improvement compared to a three-NPNV peptide. Hence, our results demonstrate proof of concept for this spatially constrained multigrafting approach but leave room for improvement. We note, however, that L9 Fabs are capable of binding to the native peptide in three similar but distinct spirals (PDB: 8EH5, 8EKA, and 8EK1), indicating a range of flexibility in the complexed native structure in which our scaffold complex may fall.

We noted that order-3 TES were more successful in generating viable in silico models compared to the other design order strategies tested, with our yeast display screen having identified no viable designs from the order-1 or order-2 strategies. This provides an important lesson for future work with de novo design of multiepitope scaffolds, namely that multiple permutations of the order of epitopes within the de novo scaffold sequence should be tested. It is also likely that increased sampling in order-3 with a larger diversity of de novo folds might have produced a larger pool of TES for subsequent yeast display screening and downselection. With these successes, it may also be prudent to increase computational resources to generate larger and more diverse libraries of backbones for single-epitope and DES as well. We also note that the designs reported here may not be the entirety of successes capable of binding. As these are linear epitopes to a high affinity antibody, the yeast libraries underwent stringent proteolytic pressure and only the highest binders were sorted for, resulting in extremely converged sequences. It is possible that with less stringent pressure, the resultant yeast display libraries could have yielded more binders with a stable scaffold fold. Indeed, none of the isolated sequences scored the highest in TM-score, pLDDT, or lowest in PAE; we postulate that sequences not so highly ranked to be selected for the top 64 yeast display screen but still passing these filters could also have yielded successful designs. Unfortunately, it is still elusive in the field of de novo protein design of any one in silico metric that has the best predictive ability for in vitro, much less in vivo, success.

Using previously established nanoparticle platforms for vaccination and a mouse model of malaria sporozoite liver invaction (33), we showed that our scaffold-based immunogens elicited antibody responses to a junctional peptide and provided a modest degree of inhibition of liver invasion. Scaffold-based nanoparticles elicited similar levels of inhibition to the J1 peptide nanoparticles that included one NPNV and one NANP epitope but lower inhibition than the J2 and J3 peptide nanoparticles that included at least two NPNV and two NANP epitopes. The J1-, J2-, and J3 peptide immunogens each include the N-terminal portion (KLKQPADGNPDP) and relatively extended portions of the native junctional sequence. These immunogens could elicit antibody responses to any linear portion of the CSP epitope within the J3 sequence as well as potential conformational epitopes sampled by J3. A limitation of the scaffold approach in this case may be in presenting a rigid conformation of a small peptide epitope. Multiple monoclonal antibodies that bind different conformations of junctional and major repeat epitopes have been described that provide protection in RTS,S vaccination (35). Nevertheless, future improvements in the scaffold design and future testing in mouse models designed to reproduce L9 immunogenetics would enable more stringent testing of the potential protective value of such scaffold-based immunogens for malaria. Next-generation scaffolds could include improved spatial placement of the NPNV 3 epitope in relation to the first two NPNV epitopes; potentially improved immuno-focusing to the NPNV epitopes via reduction of scaffold size and/or glycan masking; potentially improved nanoparticles and/or linkers between scaffolds and underlying nanoparticle to ensure sufficient space for antibodies to bind the three NPNV repeats without steric clash with other scaffolds on the nanoparticle and, following the germline-targeting strategy that has been employed to elicit precursors of different classes of HIV broadly neutralizing antibodies (bnAbs) (36–40), potential introduction of scaffold mutations to ensure efficient activation of L9 germline-precursor B cells. Analogous to developments in HIV bnAb-precursor mouse models, more advanced mouse models to assess elicitation of and liver invasion inhibition by L9-like responses could potentially include experiments with L9 inferred-germline knockin mice with or without adoptive transfer (41–44), or human Ig locus transgenic mice (45), or recombining knockin models allowing for production of diverse L9-like precursors (46).

Overall, we have demonstrated the ability of deep learning methods to scaffold arrays of linear epitopes in their preferred binding conformation and with at least two epitopes in their native relative spatial positions. We have also taken initial steps toward design of epitope scaffold immunogens that have potential to elicit L9-like antibodies to prevent malaria. Additional immunogen design optimization should be pursued, as noted above. Application of newer deep learning methods will likely further improve the success rate and applicability of the methods tested here. This work may open doors for design of scaffolds presenting more complex arrays of functional sites for diverse biological applications.

## Methods

### De Novo Design of Single-NPNV Scaffolds with RoseTTAFold Hallucination.

We designed SES using a RoseTTAFold hallucination method. This involves a sequence passed to the RoseTTAFold network to predict 3D coordinates that are scored by a motif recapitulation loss function used to then update the sequence to the next iteration of RoseTTAFold (19). Initially, 167 trajectories using template PDB:7RQQ (residues 106 to 111) were run using 600 steps of gradient descent with a repulsive loss (α = 3.5 Å, weight = 2), a coordinate RMSD loss (weight = 2) and a radius of gyration loss (threshold = 16 Å, weight = 1), sampling scaffolds between 50 and 85 residues. Out of 46 designs with AF pLDDT > 80, RoseTTAFold model motif RMSD_backbone_ to crystal structure input <1 Å, and radius of gyration <13 Å, 19 were manually selected for experimental screening. Finally, designs were subject to ProteinMPNN, a complementary, message passing neural network using backbone information and interresidue information generate sequences intended to recover the backbone fold (23).

### De Novo Design of Double- and Triple-NPNV Scaffolds with RFdiffusion.

Multi-NPNV scaffolds were designed using the RFDiffusion network which fine-tunes RoseTTAFold as a denoising network in a generative diffusion model (22). DES were designed by either scaffolding two repeats from PDB:8EH5 separately (Chain G: 107 to 110 and 115 to 118) with a 5 to 20 residue diffused linker or by including the 4-residue linker from the native structure (111 to 114). For the discrete epitope repeats, 1,170 scaffolds were generated using 20 to 50 residue sampling before the first epitope and after the second epitope, with a range of 5 to 20 residues between the two repeats of which 209 were selected using radius of gyration normalized by scaffold length < median and predicted loop composition < 110% median. Predicted loop composition was calculated as the ratio of loop residues identified by Rosetta Residue Selector Secondary Structure Selector over the total residues in the scaffold. For DES using the native linker, 10 to 40 residues were sampled before and after the motifs. 262 scaffolds out of 1,257 trajectories were selected on the same filters described above.

TES were designed using PDB:8EH5 (107 to 110, 115 to 118, and 123 to 126). 10 to 40 residues before the first repeat, 5 to 40 residues between the first and second repeat, 10 to 40 residues between the second and third repeat, and 10 to 30 residues after the third repeat were sampled. Half the trajectories were run with native repeat order (order-1) and the other half with repeat 2 and 3 swapped (order-2). Out of 949 trajectories, 76 were selected on the same filters described for DES. A third run of 500 trajectories was performed with 10 to 15 residues before repeat 3, 22 to 28 residues, then repeat 1, 22 to 28 residues, then repeat 2, then 10 to 15 residues (order-3); 26 trajectories were selected on the same filters.

Finally, seven DES with native linker designs, six DES de novo linker designs, two order-1 TES, 1 order-2 TES, and 7 order-3 TES designs were manually selected for ProteinMPNN redesign. 500 sequences were sampled at 4 sampling temperatures (0.1,0.2,0.3,0.4) for each scaffold. 8,515 double epitope scaffold designs and 4,411 TES designs with AF pLDDT > 80, PAE < 10, and TMscore to starting diffused scaffold > 0.7 were selected for yeast display screening. Designs with TMscore > 0.8 were ranked by highest pLDDT and lowest PAE, and 64 double-epitope and 64 triple-epitope designs were ordered for preliminary screening.

### Yeast Display.

A colony of EBY100 yeast cells was picked and incubated in YPD media and 1:100 PenStrep at 30 °C overnight. The next day, 1 μg of oligopools or assembled ultramers containing degenerate NNK codons from IDT amplified using Platinum™ SuperFi™ DNA Polymerase was mixed with 5 μg of yeast display vector and dried to less than 10 μL using a rotary evaporator then chilled on ice. When EBY100 cells reached an OD_600_ of 1.5, cells were pelleted at 2,000×*g* for 5 min and resuspended in 100 mM lithium acetate and 10 mM DTT to shake for 10 min at 30 °C. After making cells electroporation competent, they were pelleted at 2,000×*g* for 5 min, washed in cold water, pelleted at 2,000×*g* for 5 min, and resuspended in 500 μL aliquots of water. DNA was resuspended in 250 μL of cells and added to 2 mm electroporation cuvettes. Cells were electroporated using a Bio-Rad electroporator using square wave voltage 500 V, pulse length 15 ms, and 1 pulse. Cells were recovered in 25 mL of SD-Trp-Ura and grown for 2 d.

Transformed yeast were added to induction media SGCAA and 1:100 PenStrep at 30 °C to shake overnight. The next day, cells were pelleted at 3,000×*g* for 5 min and washed with 0.1% BSA 1X PBS twice. Then, they were digested with 2.5 μg/mL, 25 μg/mL, or 250 μg/mL of trypsin or chymotrypsin for 15 min at room temperature. After wash, cells were stained for 1 h at 4 °C. Cells were pelleted at 3,000×*g* for 5 min and washed with 0.1% BSA 1X PBS twice before staining with Alexa Fluor 647-conjugated anti-human IgG (Jackson Immunoresearch) and chicken anti-C-Myc Antibody- FITC Conjugated (ICL Antibodies CMYC-45F) for 1 h at 4 °C. Cells were pelleted at 3,000×*g* for 5 min and washed with 0.1% BSA 1X PBS twice before being resuspended in 0.1% BSA 1X PBS for FACS. Data were analyzed by gating on single cells using FSC, SSC, and width in FlowJo 10.7.1 (Beckton Dickinson). After final sort, cells were spread on the SDA-Trp-Ura plate. Colony PCR was done using the Thermo Scientific^TM^ Phire^TM^ Plant Direct PCR kit and sent for Sanger sequencing.

### DNA Gene Synthesis and Cloning.

All genes were synthesized at Genscript, Inc. Nanoparticles were cloned into pHLsec between the leader and a double stop coding using the AgeI and KpnI cloning sites. Antibody heavy chains were cloned into pCW-CHIg-hG1 between the leader and IgG1 human constant domain using EcoRI and NheI cloning sites. Kappa chains were cloned into pCW-CLIg-hk between the leader and human kappa constant region using the EcoRI and BsiWI. Lambda chains were cloned into pCW-CLIg-hL2 between the leader sequence and the human lambda constant region using the EcoRI and AvrII cloning sites.

### Protein Purification.

DNA was transfected with a plasmid into FreeStyle 293F cells (Invitrogen, Cat No. R79007) using 293Fectin (ThermoFisher) and proteins were expressed at 37 °C for 4 d. Monomers were expressed with C-terminal His-tags purified by nickel columns followed by gel-filtration using a Superdex 200 Increase size-exclusion chromatography column (GE Healthcare).

### Circular Dichroism.

Proteins were buffer exchanged into 10 mM sodium phosphate and diluted to 400 μL of 0.5 mg/mL. Samples were loaded into quartz cuvettes and analyzed on Jasco J815 from 260 nm to 195 nm per the manufacturer’s instructions.

### Differential Scanning Calorimetry.

Proteins were buffer exchanged into 10 mM sodium phosphate and diluted to 400 μL of 0.5 mg/mL. Samples were prepared in a 96-well deep well plate and paired with buffer only wells. Two pairs of buffer–buffer wells were run between samples to ensure wash. Instrument “VP-Cap DSC” made by MicroCal LLC was operated according to the manufacturer’s instructions with scanning range from 20 to 110 °C.

### Surface Plasmon Resonance.

Kinetics and affinities of Fab/antigen interactions were measured on a Biacore 8 K (Cytiva) using CM3 Sensor Chip (Cytiva). We used 1x HBS-EP+ pH 7.4 running buffer (20× stock from Teknova, Cat. No H8022) supplemented with BSA at 1 mg/mL. We amine coupled about 17 to 30 RUs of antigen. The coupling buffer was 10 mM Sodium Acetate pH 4.5. Fab analyte was passed over the flow cell at 30 μL/min for 2 min followed by a 10 min dissociation time without regeneration. Raw sensograms were analyzed using Biacore Insight Evaluation software, version4.0.8.20368 (Cytiva), including reference spot and blank double referencing, and both Equilibrium and Kinetic fits with Langmuir model.

### SECMALS.

The molecular weights of the antigens were assessed by size-exclusion chromatography–multiangle light scattering (SEC-MALS) in PBS using a Superdex 75 Increase 10/300 GL column (Cytiva) operating with an isocratic flow of 0.5 mL/min followed by DAWN HELEOS II and Optilab T-rEX detectors (Wyatt Technology).

### Kinetic Exclusion Assay.

KDs were measured using the n-Curve KinExA Analysis method provided in KinExA Pro software and described in Technology Note 229. To perform KinExA equilibrium experiments, the following materials were prepared: Constant Binding Partner (CBP), human IgG, was produced in Schief Lab; antigen was expressed and purified in Schief Lab; labeling antibody for IgG was Alexa Fluor 647 conjugated AffiniPure Goat Anti-Human IgG, Fc Fragment Specific, Jackson ImmunoResearch code 109-605-008, 500 ng/mL in running buffer (prepared fresh from 1,000× concentrated stock that is stored at −80 °C); running buffer was the same as sample buffer and it was 1× HBS-EP+ pH 7.4 (20× stock from Teknova, Cat. No H8022) supplemented with BSA from Sigma (cat. A3294) at 1 mg/mL; beads were Thermo Scientific UltraLink azlactone-activated, beaded-polyacrylamide resin cat. number 53110 and antigen was used as a cross-linked capturing reagent; and the instrument was KinExA 3200 with Autosampler model SAPIDYNE-AIM3300 (Sapidyne Instruments).

All measurements and equilibrations were run at room temperature (about 25 °C). The antigen was serially diluted into samples having constant concentration of antibody/Fab. All samples were equilibrated for 24 h before the start of the measurement. All data points were measured in duplicates. Data were analyzed using n-curve function of the KinExA Pro software (Version 4.0.12).

### Modeling.

Models of novel proteins were generated using Alphafold2 (27, 47).

### X-Ray Crystallography.

For crystallization, L9 Fab was expressed in ExpiCHO cells and purified using a HiTrap Protein G HP column (GE Healthcare), followed by size exclusion chromatography in 1X TBS buffer, pH 7.8. The complex of L9 Fab and SES-2 scaffold was prepared by mixing the Fab and scaffold in a 1:1 molar ratio. Crystallization screening was performed at 10 mg/mL using the sitting drop vapor diffusion method with our high-throughput CrystalMation system (Rigaku) at The Scripps Research Institute. Crystals grew in a solution containing 0.08 M sodium cacodylate (pH 6.5), 0.16 M calcium acetate, 20% glycerol, and 14.4% (wt/vol) PEG8000, and appeared within 14 d at 293 K. Diffraction data were collected at Stanford Synchrotron Radiation Lightsource (SSRL), beamline 12-1. The data were processed and scaled using the HKL2000 package (48). The structure was determined by molecular replacement using Phaser (49), with the Fab L9 structure (PDB ID: 7RQR) as a search model. The SES-2 scaffold was manually fitted into the observed electron density, followed by multiple rounds of refinement using phenix.refine (50), with iterative model building in Coot (51).

### Cryo-EM Data Collection and Model Building.

Purified complex was diluted to 0.1 mg/mL in TBS and 3.5 μL of the sample was applied to graphene oxide grids, blotted for 2.5 s at 100% humidity, 4 °C, and plunge frozen in liquid ethane with a Vitrobot Mark IV (Thermofisher Scientific). Data were collected on a 200 kV Thermo Fisher Scientific Glacios (II) with a Thermo Scientific Falcon 4i Direct Electron Detector using EPU Software. Nominal magnification was 190,000x with a pixel size of 0.718 Å in a defocus range of −0.9 to −1.8 μm and an average dose of 45 e^−^/Å^2^ per micrograph. Image motion correction and CTF estimations were performed with CryoSPARC Live software before transferring to cryoSPARC v4.3.1 (52). Particles were picked with a combination of Manual Picker and Template Picker and subjected to iterative rounds of 2D classification. 623 k particles corresponding to good 2D classes were subjected to Ab Initio 3D modeling and Heterogenous Refinement. 370 k particles corresponding to the best heterogenous class were refined using Non-Uniform Refinement (with global CTF corrections) to obtain cryo-EM map resolution of 3.1 Å based on Gold-Standard Fourier Shell Correlation (GSFSC at 0.143).

An initial model of the scaffold generated using Alphafold 2 (27) and L9 Fab models (PDBid : 8EH5) was fitted into the cryo-EM map in ChimeraX v1.7 (53). The complete model was then refined iteratively with Coot v9.8.7 (54), Phenix real space refine (55), and Rosetta Relax (56). Final validation was performed with the MolProbity (57) and EMRinger (58) and the statistics are reported in *SI Appendix*, Table S2.

### ELISA.

ELISA buffers were made as follows: washing buffer (PBS + 0.2% tween 20), blocking buffer (5% skim milk in PBS with 1% FBS and 0.2% tween 20), dilution buffer (PBS with 1% FBS and 0.2% tween 20), and stop solution (340 mL of dH2O with 10 mL of sulfuric acid). 96-well Corning costar assay plates were coated with 25 μL of antigen at 2 μg/mL in PBS overnight at 4 °C. After each incubation, plates were washed three times. Blocking buffer was added, incubating at room temperature for 1 h. Sera were diluted threefold from a starting dilution of 1:500 and incubated for 1 h. Antibodies were diluted fourfold from a starting concentration of 10 μg/mL and incubated for 1 h. 25 μL of peroxidase secondary antibody was added for 1 h incubation. A 1:5 dilution of TMB was added to each plate at 25 μL per well. Plates were left to stand for approximately 4 min before 25 μL per well of stop solution was added. A plate reader reads at 450 and 570 nm, with the delta between the two readings measured and analyzed. Area under the curve (AUC) was calculated using Prism 9.1.0.

### Statistics.

Data were analyzed and plotted using Prism 10.1.0 (Graphpad). Medians and interquartile ranges were shown when appropriate. All measurements were taken using discrete samples. Statistical analysis was performed using Prism software using a two-tailed Mann–Whitney test assuming non-normal distribution. *P* values below 0.05 (*), 0.005 (**), and 0.0005 (***) were considered significant and indicated by asterisks.

### Mouse Immunizations.

Our standard two-dose schedule utilized female C57BL/6J mice that were 7 to 8 wk old at the time of the first immunization. Mice were immunized at week 0 and week 5 with 0.5 nanomoles of immunogen and 5 μg of SMNP adjuvant, SQ scruff of the neck. Mice were bled 1 wk before the first immunization and 1 wk after the second immunization for 100 μL of blood.

### Transgenic Sporozoite Challenge.

At 2 wk after last immunization, all mice were challenged through retro-orbital injection with *P. berghei* transgenic sporozoites expressing *P. falciparum* CSP and luciferase. Forty-two hours after challenge, mice were injected IP with 100 μL of D-luciferin (30 mg/mL), having been anesthetized by exposure to isoflurane. Bioluminescence in the liver was measured using an AMI HTX (Spectral Instruments). Flux values were normalized using the below formula.

$$\begin{array}{c} \%Inhibition=100-\left(\left(\frac{Flux_{immunogen}}{Flux_{adjuvantonly}}\right)*100\right). \end{array}$$

## Ethics Statement

All the experimental animal work was performed in strict compliance to the guidelines of Scripps Institutional Animal Care and Use Committee, who approved this study (under animal use protocol authorization 15-0003-4 and 22-0007-1).

## Supplementary Material

Appendix 01 (PDF)

## Data Availability

The crystal structure of SES-2 bound to L9 Fab was deposited in the Protein Data Bank with PDBid of 9D3J (26). The cryo-EM map and coordinates for TES01 bound to three L9 Fabs were deposited in the Electron Microscopy Data Bank under accession number EMD-45640 (30) and the Protein Data Bank with PDBid of 9CK4 (29). All other data are included in the manuscript and/or supporting information.
