# Supplementary material for: Deep learning–enabled scaffolding of spatial arrays of PfCSP epitopes
Source: Proc Natl Acad Sci U S A. 2026 Apr 7;123(15):e2521914123. doi: 10.1073/pnas.2521914123 (PMC13079917; doi:10.1073/pnas.2521914123)
Supplement: Supplementary file 1 — Appendix 01 (PDF) [file pnas.2521914123.sapp.pdf]

PNAS

Supporting Information for
Deep learning-enabled scaffolding of spatial arrays of PfCSP epitopes

Nelson R. Wu[1,*], Karla M. Castro[2,*], Nathan Beutler[1,*], Wen-Hsin Lee[3], Sai S.R. Raghavan[3], Gregory M. Martin[3], Monika Jain[3], Sashank Agrawal[3], Alessia Liguori[1,4], Oleksandr Kalyuzhniy[1,4], Patrick D. Skog[1,4], Sierra Terada[1,4], Yen-Chung Lai[1,5], Justin Ndihokubwayo[1,5], Danny Lu[1,4], Saman Eskandarzadeh[1,4], Nushin Alavi[1,4], Nicole Phelps[1,4], Ryan Tingle[1,4], John E. Youhanna[1,4], Sonya Amirzehni[1,4], Thomas F. Rogers[1,5], Dennis R. Burton[1,4,6], Ian A. Wilson[3,7], Andrew B. Ward[3], Bruno E. Correia[2,†], William R. Schief[1,4,6,8,†]

[1]Department of Immunology and Microbiology, The Scripps Research Institute, La Jolla, CA 92037, USA
[2]Institute of Bioengineering, École Polytechnique Fédérale de Lausanne, Lausanne, Switzerland; Swiss Institute of Bioinformatics (SIB), Lausanne, Switzerland.
[3]Department of Integrative Structural and Computational Biology, The Scripps Research Institute, La Jolla, CA, 92037, USA.
[4]IAVI Neutralizing Antibody Center, The Scripps Research Institute, La Jolla, CA 92037, USA
[5]Division of Infectious Diseases, Department of Medicine, University of California, San Diego, La Jolla, CA 92037, USA.
[6]The Ragon Institute of Massachusetts General Hospital, Massachusetts Institute of Technology and Harvard University, Cambridge, MA 02139, USA
[7]The Skaggs Institute for Chemical Biology, The Scripps Research Institute, La Jolla, CA, 92037, USA.
[8]Moderna, Inc., Cambridge, MA 02139, USA

Correspondence to: William Schief (schief@scripps.edu) or Bruno Correia (bruno.correia@epfl.ch)

This PDF file includes:

Figures S1 to S14
Tables S1 to S3

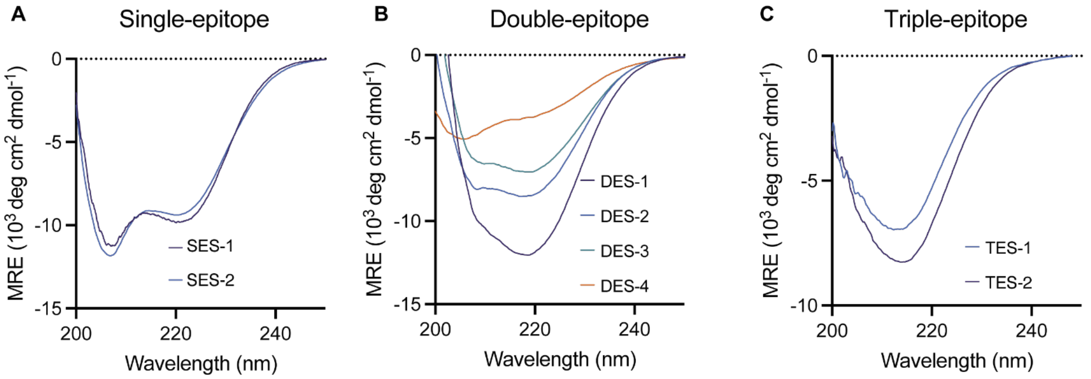

Fig. S1. Characterization of single- double- and triple-scaffolds.

A) CD spectra of single-epitope scaffolds. B) CD spectra of double-epitope scaffolds. C) CD spectra of triple-epitope scaffolds.

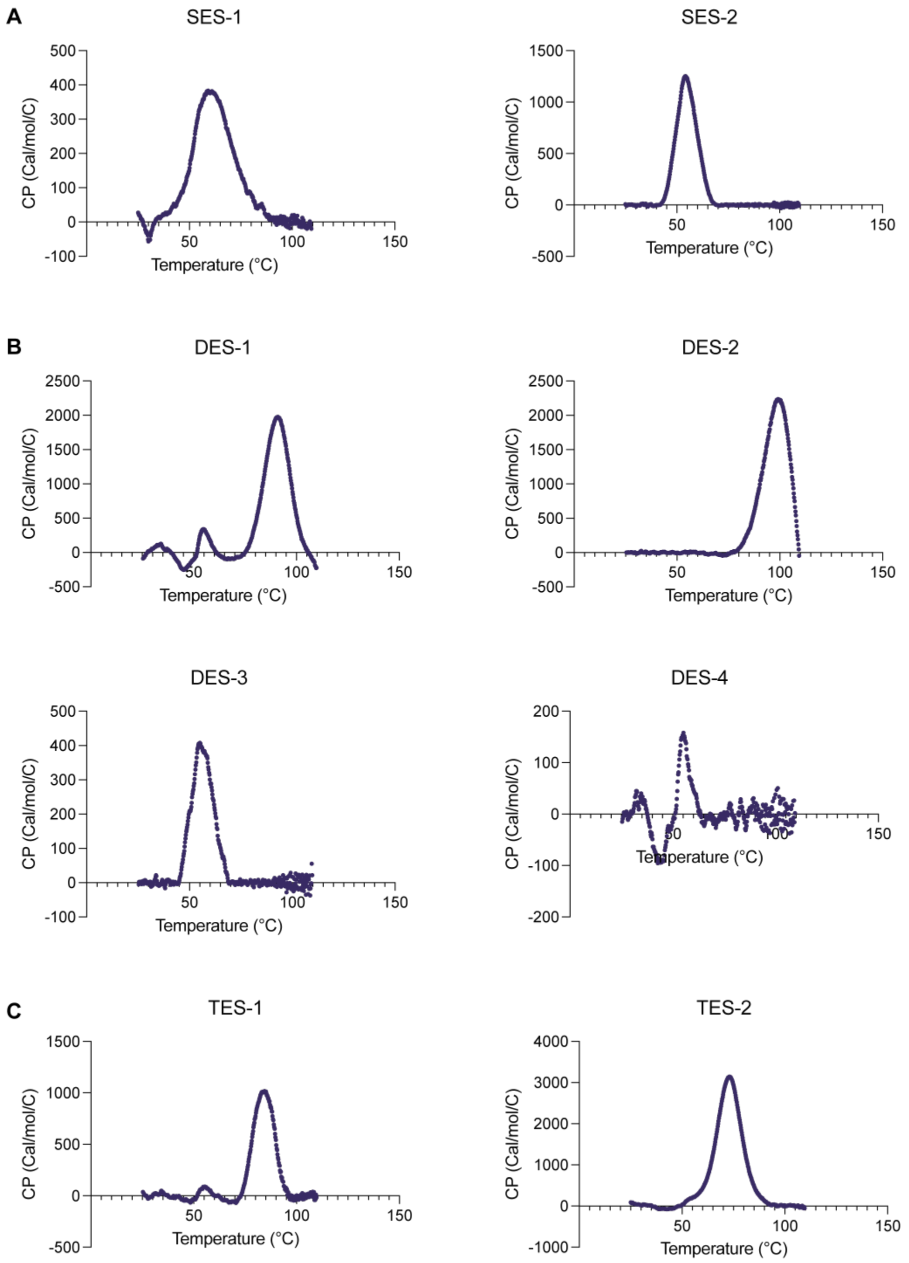

Fig. S2. Thermal stability of epitope scaffolds.

A) DSC melting curve of single-epitope scaffolds. B) DSC melting curve of double-epitope scaffolds. C) DSC melting curve of triple-epitope scaffolds.

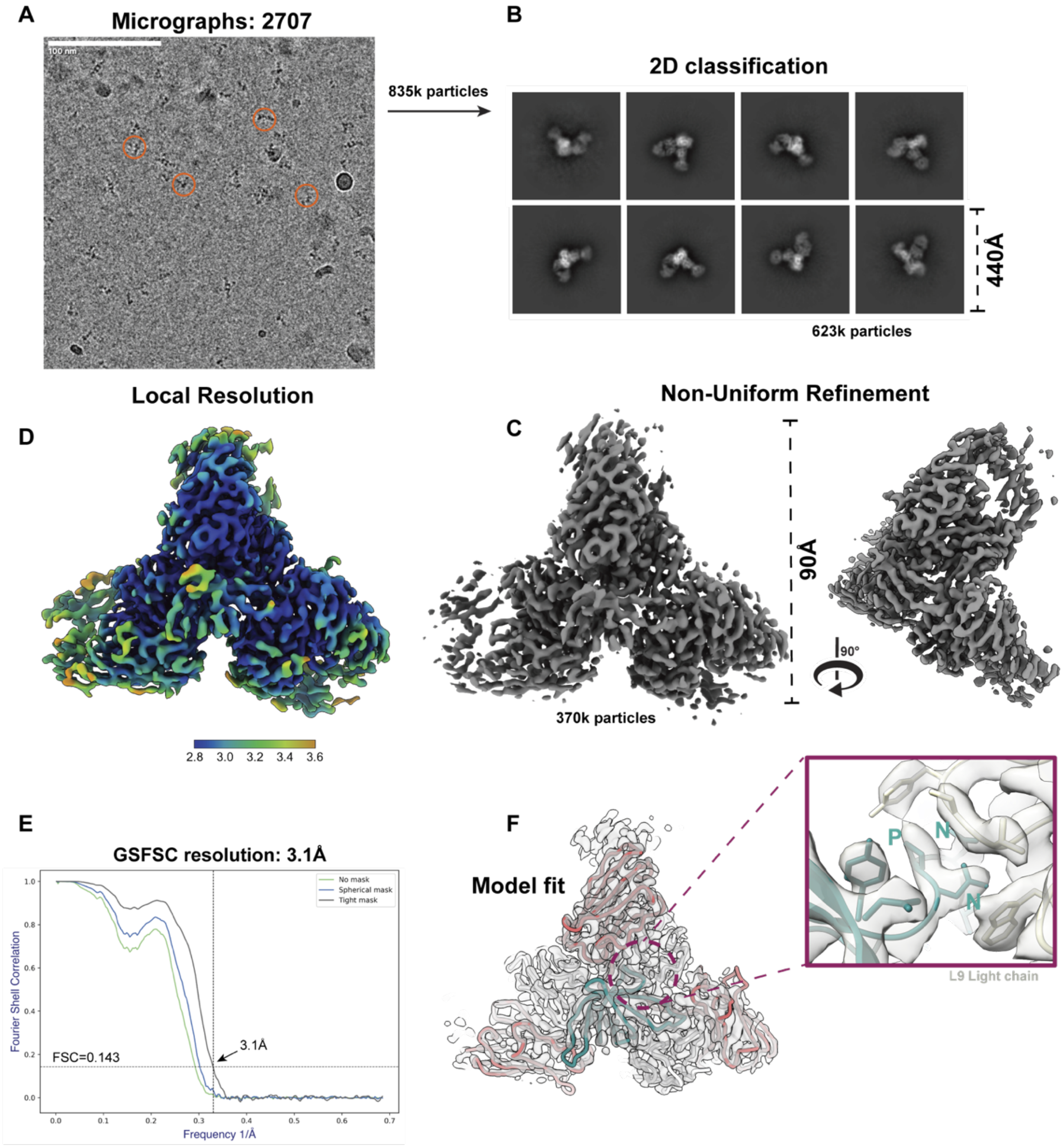

Fig. S3. Cryo-EM structure and resolution.

A) Representative micrograph on a graphene oxide grid. B) Representative 2D class averages. C) Non-Uniform refinement of the map generated using the final particle stack of ~370k. D) Local resolution map of the final consensus reconstruction after post-process CTF refinement and sharpening. E) Fourier Shell Correlation (FSC) plot of map in D. F) Overlay of the final structure with the final consensus cryoEM map.

A

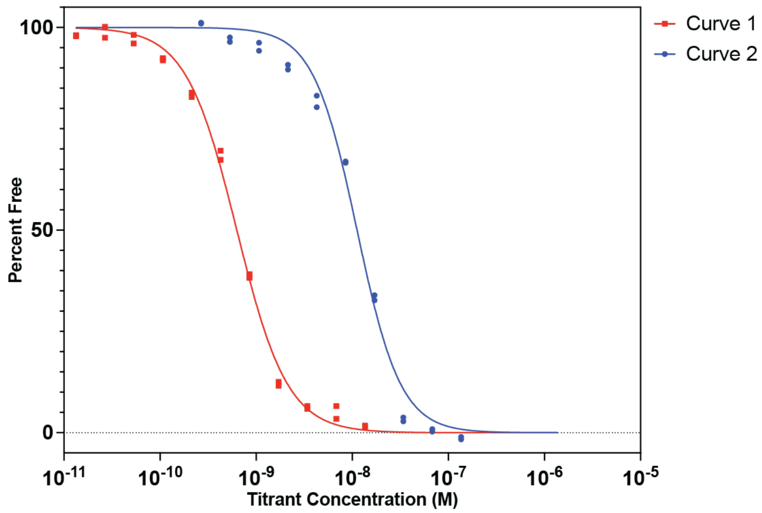

TES-1	Curve 1	Curve 2
Nominal CBP (M)	2.48E-08	1.24E-09
Ratio	291.180	14.583
Sig 100%	1.196	1.577
NSB	0.092	0.152

B

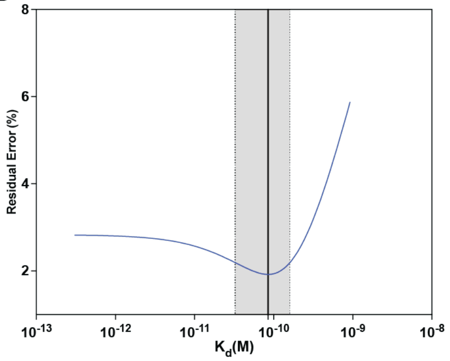

TES-1		95% confidence interval	
	Overall	High	Low
Kd (M)	8.50E-11	1.60E-10	3.27E-11
% Error	1.9		

C

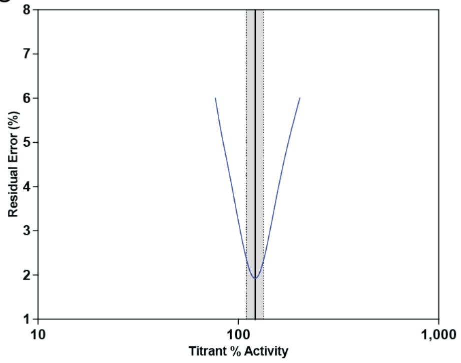

TES-1		95% confidence interval	
	Overall	High	Low
Titrant % Activity	121.3	134.0	109.8

Fig. S4. Binding of TES-1 to L9 IgG by KinExA.

A) Titrations of TES-1 into buffer of constant L9 IgG at 2.48E-08 (Curve 1) and 1.24E-09 (Curve 2) were equilibrated. Percent free L9 IgG was calculated and fit using an equilibrium model. Experiments were run in technical replicates. Data points represent a single technical replicate.
B) The residual error in the best fit of the Kd was plotted with a solid line identifying the overall Kd and dotted lines showing the 95% confidence interval.
C) The residual error in the best fit of the titrant % activity was plotted with a solid line identifying the overall titrant % activity and dotted lines showing the 95% confidence interval.

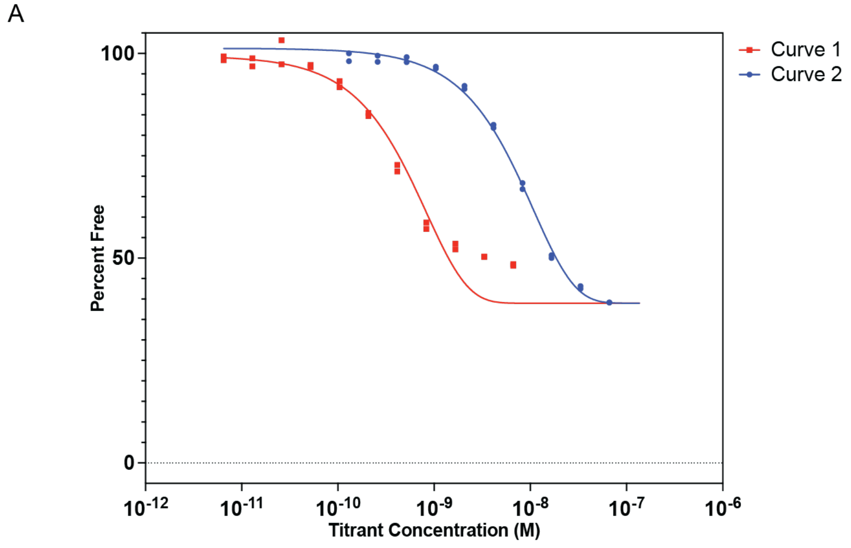

TES-2	Curve 1	Curve 2
Nominal CBP (M)	2.48E-08	1.24E-09
Ratio	7.939	0.398
Sig 100%	1.296	1.512
NSB	-0.592	0.083

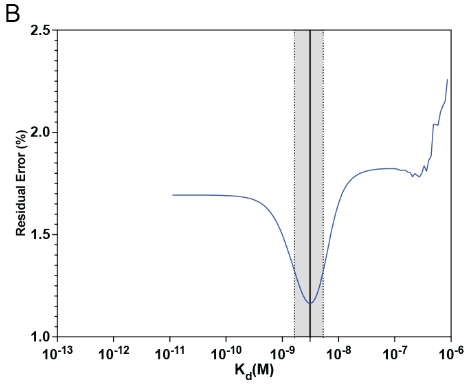

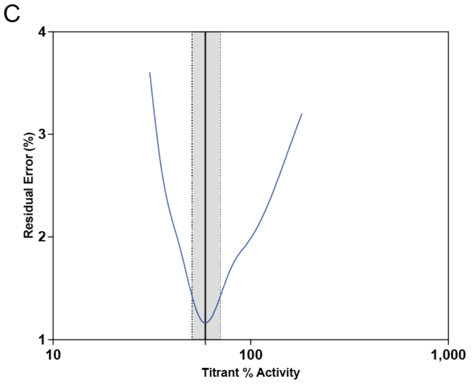

TES-2		95% confidence interval	
	Overall	High	Low
Effective Kd	3.12E-09	5.38E-09	1.63E-09
% Error	1.2		

TES-2		95% confidence interval	
	Overall	High	Low
Titrant % Activity	58.9	70.2	50.5

Fig. S5. Binding of TES-2 to L9 IgG by KinExA.

A) Titrations of TES-2 into buffer of constant L9 IgG at 2.48E-08 (Curve 1) and 1.24E-09 (Curve 2) were equilibrated. Percent free L9 IgG was calculated and fit using an cooperativity model. Experiments were run in technical replicates. Data points represent a single technical replicate.
B) The residual error in the best fit of the Kd was plotted with a solid line identifying the overall Kd and dotted lines showing the 95% confidence interval.
C) The residual error in the best fit of the titrant % activity was plotted with a solid line identifying the overall titrant % activity and dotted lines showing the 95% confidence interval.

A

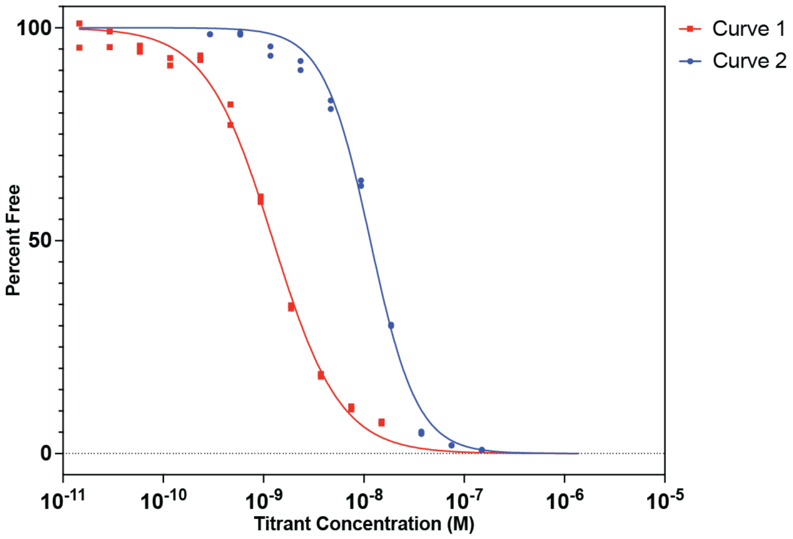

DES-1	Curve 1	Curve 2
Nominal CBP (M)	2.48E-08	1.24E-09
Ratio	38.433	1.925
Sig 100%	1.410	1.478
NSB	0.026	0.083

B

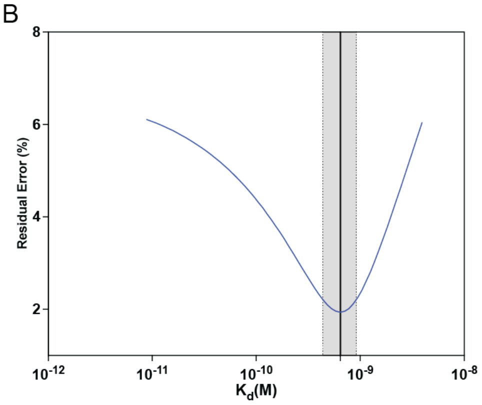

DES-1		95% confidence interval	
	Overall	High	Low
Kd (M)	6.44E-10	9.18E-10	4.37E-10
% Error	1.9		

C

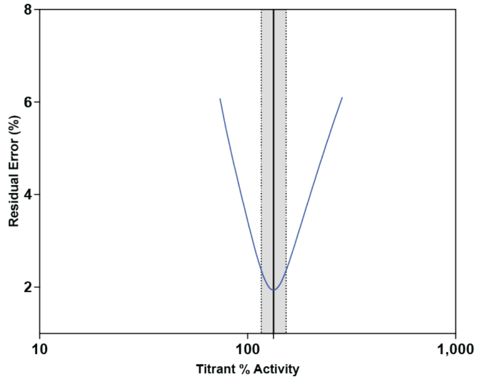

DES-1		95% confidence interval	
	Overall	High	Low
Titrant % Activity	133.1	153.4	116.2

Fig. S6. Binding of DES-1 to L9 IgG by KinExA.

A) Titrations of DES-1 into buffer of constant L9 IgG at 2.48E-08 (Curve 1) and 1.24E-09 (Curve 2) were equilibrated. Percent free L9 IgG was calculated and fit using an equilibrium model. Experiments were run in technical replicates. Data points represent a single technical replicate.
B) The residual error in the best fit of the Kd was plotted with a solid line identifying the overall Kd and dotted lines showing the 95% confidence interval.
C) The residual error in the best fit of the titrant % activity was plotted with a solid line identifying the overall titrant % activity and dotted lines showing the 95% confidence interval.

A

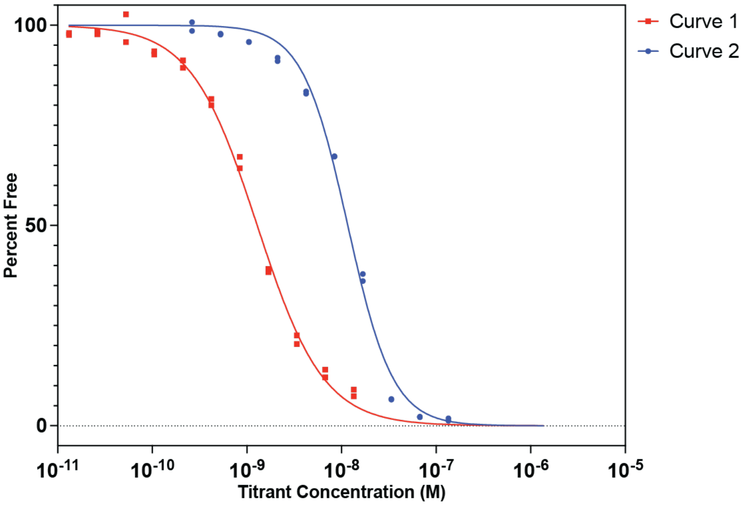

DES-2	Curve 1	Curve 2
Nominal CBP (M)	2.48E-08	1.24E-09
Ratio	35.682	1.787
Sig 100%	1.437	1.423
NSB	0.023	0.072

B

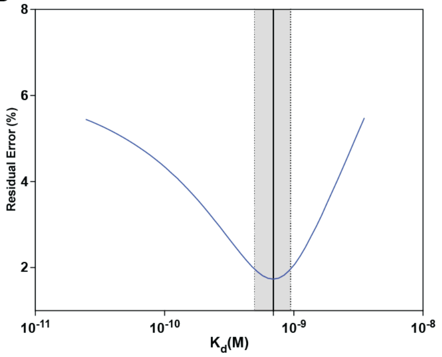

DES-2		95% confidence interval	
	Overall	High	Low
Kd (M)	6.94E-10	9.47E-10	4.95E-10
% Error	1.7		

C

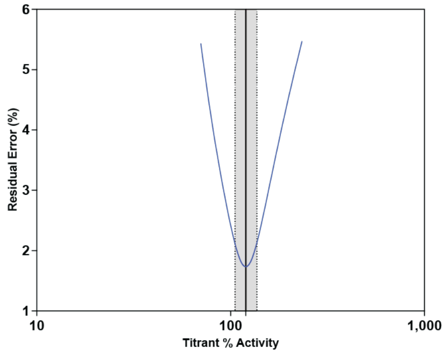

DES-2		95% confidence interval	
	Overall	High	Low
Titrant % Activity	119.8	136.4	105.2

Fig. S7. Binding of DES-2 to L9 IgG by KinExA.

A) Titrations of DES-2 into buffer of constant L9 IgG at 2.48E-08 (Curve 1) and 1.24E-09 (Curve 2) were equilibrated. Percent free L9 IgG was calculated and fit using an equilibrium model. Experiments were run in technical replicates. Data points represent a single technical replicate.
B) The residual error in the best fit of the Kd was plotted with a solid line identifying the overall Kd and dotted lines showing the 95% confidence interval.
C) The residual error in the best fit of the titrant % activity was plotted with a solid line identifying the overall titrant % activity and dotted lines showing the 95% confidence interval.

A

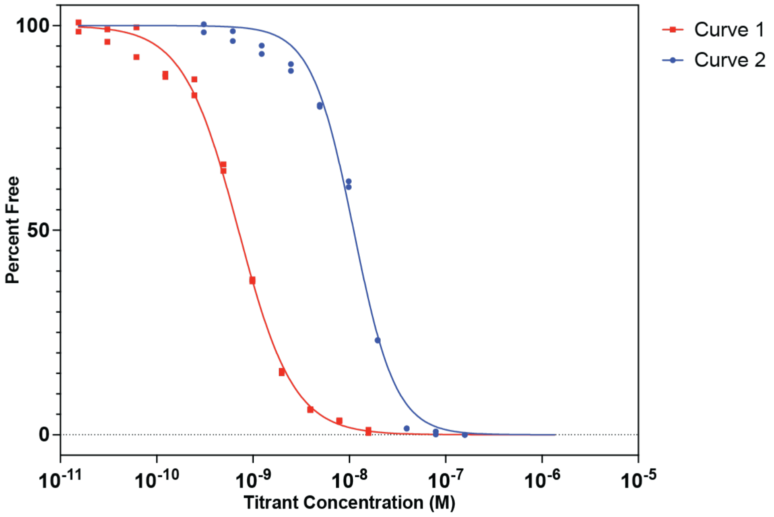

DES-3	Curve 1	Curve 2
Nominal CBP (M)	2.48E-08	1.24E-09
Ratio	164.105	8.219
Sig 100%	1.449	1.473
NSB	0.017	0.125

B

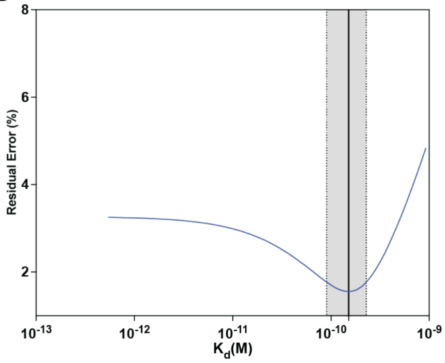

DES-3		95% confidence interval	
	Overall	High	Low
Kd (M)	1.51E-10	2.29E-10	9.04E-11
% Error	1.6		

C

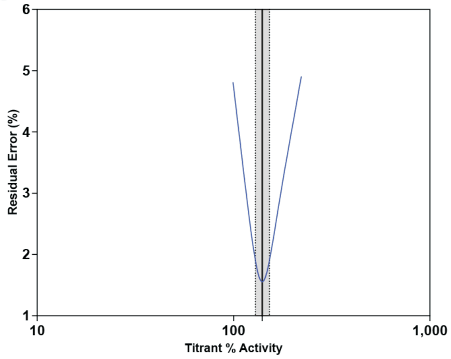

DES-3		95% confidence interval	
	Overall	High	Low
Titrant % Activity	140.1	152.3	129.3

Fig. S8. Binding of DES-3 to L9 IgG by KinExA.

A) Titrations of DES-3 into buffer of constant L9 IgG at 2.48E-08 (Curve 1) and 1.24E-09 (Curve 2) were equilibrated. Percent free L9 IgG was calculated and fit using an equilibrium model. Experiments were run in technical replicates. Data points represent a single technical replicate.
B) The residual error in the best fit of the Kd was plotted with a solid line identifying the overall Kd and dotted lines showing the 95% confidence interval.
C) The residual error in the best fit of the titrant % activity was plotted with a solid line identifying the overall titrant % activity and dotted lines showing the 95% confidence interval.

A

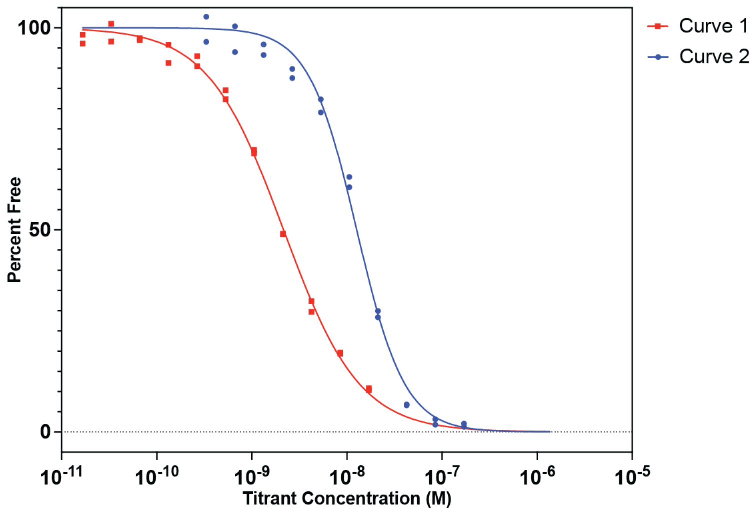

DES-4	Curve 1	Curve 2
Nominal CBP (M)	2.48E-08	1.24E-09
Ratio	15.886	0.796
Sig 100%	1.522	0.689
NSB	-0.009	0.060

B

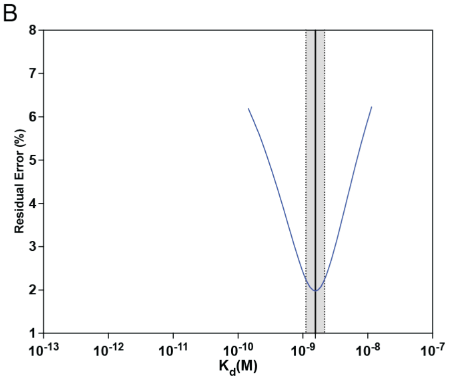

DES-4		95% confidence interval	
	Overall	High	Low
Kd (M)	1.56E-09	2.17E-09	1.11E-09
% Error	2.0		

C

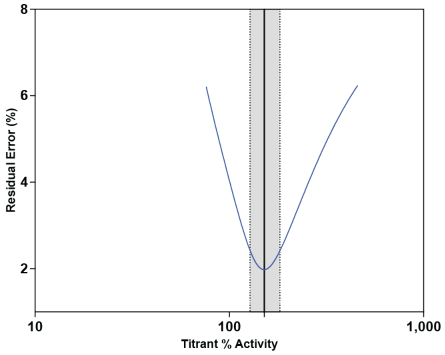

DES-4		95% confidence interval	
	Overall	High	Low
Titrant % Activity	151.1	182.3	127.8

Fig. S9. Binding of DES-4 to L9 IgG by KinExA.

A) Titrations of DES-4 into buffer of constant L9 IgG at 2.48E-08 (Curve 1) and 1.24E-09 (Curve 2) were equilibrated. Percent free L9 IgG was calculated and fit using an equilibrium model. Experiments were run in technical replicates. Data points represent a single technical replicate.
B) The residual error in the best fit of the Kd was plotted with a solid line identifying the overall Kd and dotted lines showing the 95% confidence interval.
C) The residual error in the best fit of the titrant % activity was plotted with a solid line identifying the overall titrant % activity and dotted lines showing the 95% confidence interval.

A

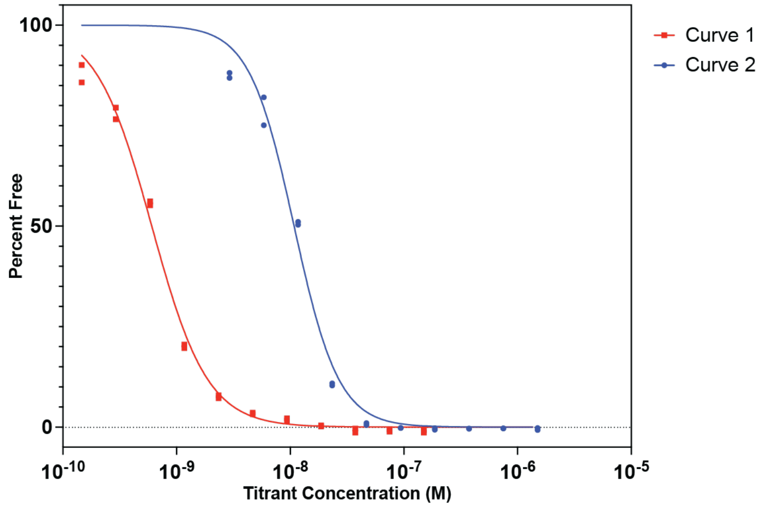

Three-NPNV peptide	Curve 1	Curve 2
Nominal CBP (M)	2.48E-08	1.24E-09
Ratio	421.041	21.086
Sig 100%	1.172	1.467
NSB	0.030	0.109

B

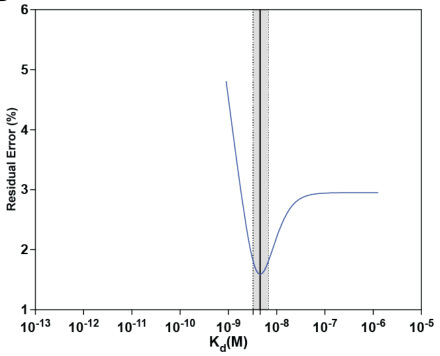

Two-NPNV peptide		95% confidence interval	
	Overall	High	Low
Kd (M)	4.56E-09	6.78E-09	3.27E-09
% Error	1.6		

C

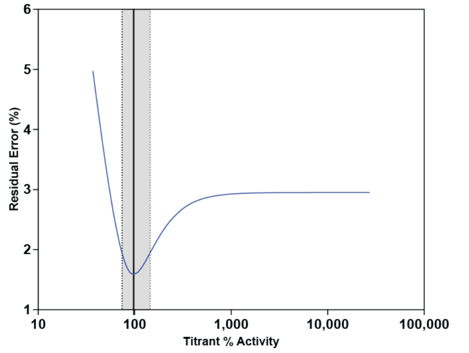

Two-NPNV peptide		95% confidence interval	
	Overall	High	Low
Titrant % Activity	97.7	144.7	73.9

Fig. S10. Binding of Three NPNV Peptide to L9 IgG by KinExA.

A) Titrations of Three NPNV Peptide into buffer of constant L9 IgG at 2.48E-08 (Curve 1) and 1.24E-09 (Curve 2) were equilibrated. Percent free L9 IgG was calculated and fit using an equilibrium model. Experiments were run in technical replicates. Data points represent a single technical replicate.

B) The residual error in the best fit of the Kd was plotted with a solid line identifying the overall Kd and dotted lines showing the 95% confidence interval.

C) The residual error in the best fit of the titrant % activity was plotted with a solid line identifying the overall titrant % activity and dotted lines showing the 95% confidence interval.

A

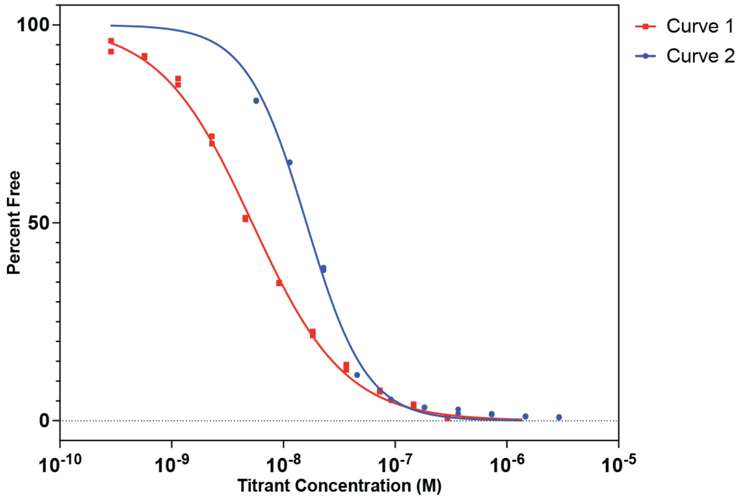

Two-NPNV peptide	Curve 1	Curve 2
Nominal CBP (M)	2.48E-08	1.24E-09
Ratio	5.436	0.272
Sig 100%	1.228	1.427
NSB	0.024	0.118

B

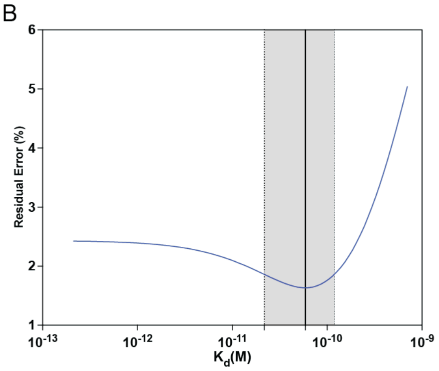

Three-NPNV peptide		95% confidence interval	
	Overall	High	Low
Kd (M)	5.88E-11	1.19E-10	2.17E-11
% Error	1.6		

C

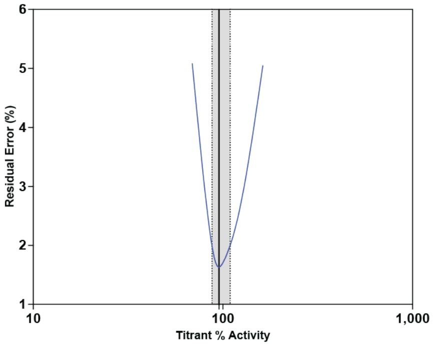

Three-NPNV peptide		95% confidence interval	
	Overall	High	Low
Titrant % Activity	95.3	109.1	87.5

Fig. S11. Binding of Two NPNV Peptide to L9 IgG by KinExA.

A) Titrations of Two NPNV Peptide into buffer of constant L9 IgG at 2.48E-08 (Curve 1) and 1.24E-09 (Curve 2) were equilibrated. Percent free L9 IgG was calculated and fit using an equilibrium model. Experiments were run in technical replicates. Data points represent a single technical replicate.

B) The residual error in the best fit of the Kd was plotted with a solid line identifying the overall Kd and dotted lines showing the 95% confidence interval.

C) The residual error in the best fit of the titrant % activity was plotted with a solid line identifying the overall titrant % activity and dotted lines showing the 95% confidence interval.

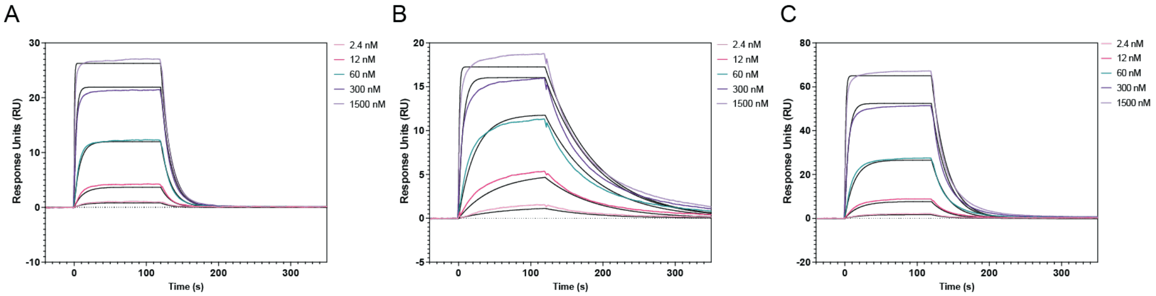

SES-1	
ka (1/Ms)	9.3E+05
kd (1/s)	7.3E-02
KD (M)	7.8E-08
Steady State KD(M)	8.4E-08

SES-2	
ka (1/Ms)	4.9E+05
kd (1/s)	1.4E-02
KD (M)	2.9E-08
Steady State KD(M)	4.2E-08

SES-2_E30A	
ka (1/Ms)	4.9E+05
kd (1/s)	4.7E-02
KD (M)	9.6E-08
Steady State KD(M)	1.1E-07

Fig. S12. Binding of Scaffolds to L9 Fab by SPR.

Fab-antigen binding kinetics and affinities were evaluated using the Biacore 8K (Cytiva) with CM3 sensor chips (Cytiva). Scaffolds were amine coupled to the chip surface. Injections of various concentrations of L9 Fab were then performed. Fitted curves are shown in black.

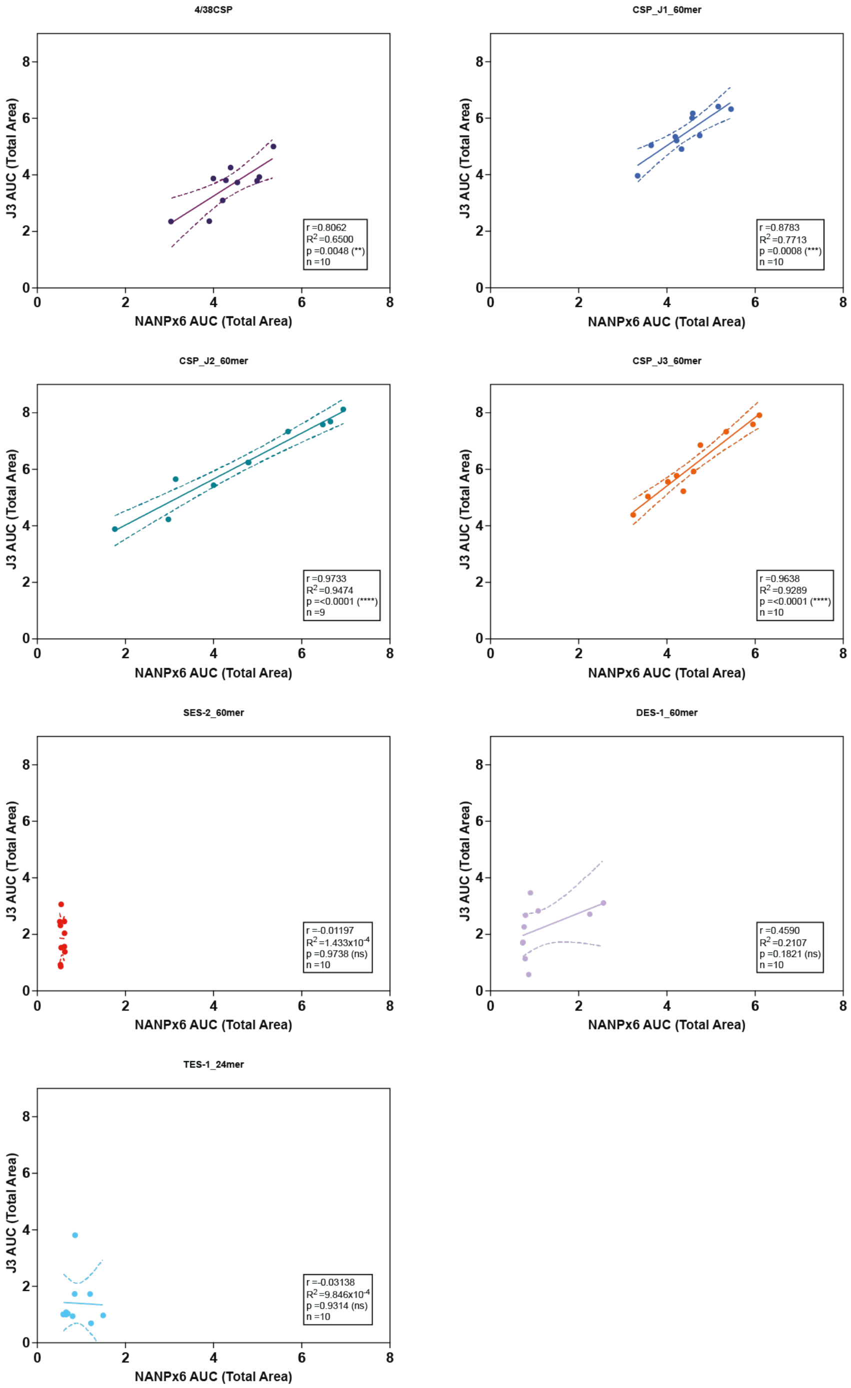

Fig. S13. Correlation of relative antibody titers to the major repeat region and junctional region following vaccination

ELISA plates were coated with a peptide composed of six sequential NANP repeats (NANPx6) or a peptide composed of three sequential junctional motifs (J3), and incubated with sera. Area under the curve was calculated for each animal and antigen. Points represent individual animals. Scatter plot showing the relationship between IgG binding to the major repeat peptide (NANPx6) and the junctional peptide (J3) in sera from immunized animals. A linear regression line with 95% confidence band is shown. Pearson correlation analysis statistics are shown for each group.

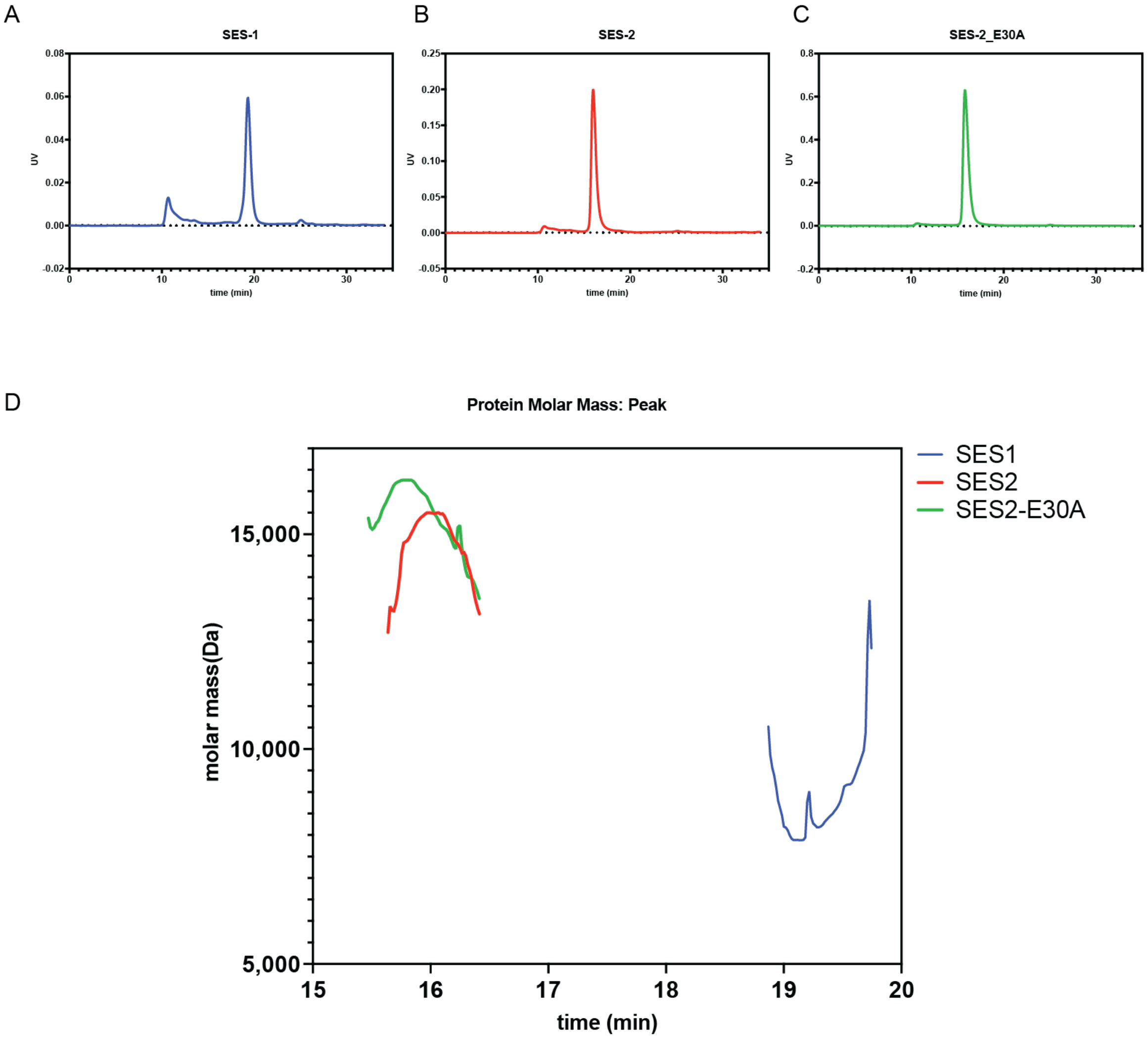

Fig. S14. SEC-MALS analysis of Single Epitope Scaffolds

Size exclusion chromatography with multi-angle light scattering analysis showed that SES-1 (expected MW 7.17 kDa) was a soluble monomer while SES-2 (expected MW 7.86 kDa) and SES-2 E30A (expected MW (7.81 kDa) were soluble dimers.

Table S1. Crystal structure data collection and refinement statistics.

	SES-2 L9-Fab complex
**Data Collection**	
Wavelength (Å)	0.979
Resolution range (Å)	27.00 - 3.50 (3.56 - 3.50)
Space group	$P\ 4_3\ 2_1\ 2$
Unit cell<br>a, b, c (Å)<br>α, β, γ (°)	 119.94 119.94 217.70<br>90 90 90
Total reflections	204,048
Unique reflections	20,805
Multiplicity	9.8 (8.4)
Completeness (%)	99.9 (99.4)
Mean I/sigma(I)	15.9 (1.2)
Wilson B (Å^2)	124
R_{merge}	0.13 (1.28)
R_{meas}	0.14 (1.36)
R_{pim}	0.04 (0.46)
$CC_{1/2}$	0.997 (0.601)
**Refinement**	
Resolution range (Å)	26.90 - 3.50
Reflections used in refinement	20,708
Reflections used for R_{free}	2000
R_{work}	0.27 (0.33)
R_{free}	0.32 (0.35)
Number of non-hydrogen atoms	6469
macromolecules	6469
solvent	-
Protein residues	852
RMS(bonds, Å))	0.002
RMS(angles, º)	0.55
Ramachandran favored (%)	92.14
Ramachandran allowed (%)	7.62
Ramachandran outliers (%)	0.25
Rotamer outliers (%)	0.28
Clashscore	7.76
Average B-value (Å^2)	140
**PDB Code**	**9D3J**

Table S2. Cryo-EM structure collection and refinement statistics.

	TES-1 scaffold complex with L9 Fabs<br>(EMDB-45640)<br>(PDB 9CK4)
**Data collection and processing**	
Magnification	190000x
Voltage (kV)	200
Electron exposure ($e^{-}/Å^2$)	45
Defocus range (μm)	-1.8 to -0.8
Pixel size (Å)	0.718
Symmetry imposed	C1
Initial particle images (no.)	835,795
Final particle images (no.)	370,307
Map resolution (Å)<br>FSC threshold (0.143)	 3.1
Map resolution range (Å)	8.1- 2.8
**Refinement**	
Initial model used (PDB code)	8EH5
Model resolution (Å)<br>FSC threshold (0/0.143/0.5)	 2.9/3.0/3.3
Map sharpening B factor ($Å^2$)	-97.6
Model composition<br>Non-hydrogen atoms<br>Protein residues<br>Ligands	 5942<br>758<br>0
Map Correlation Coefficient	0.76
R.m.s. deviations<br>Bond lengths (Å)<br>Bond angles (°)	 0.006<br>0.99
Validation<br>MolProbity score<br>Clashscore<br>Poor rotamers (%)<br>EMRinger Score<br>Cβ outliers (%)	 1.8<br>6.3<br>0.15<br>4.4<br>0
Ramachandran plot<br>Favored (%)<br>Allowed (%)<br>Disallowed (%)	 93.02<br>6.85<br>0.13

Table S3. antigen sequences.

Name	Sequence
SES-1	AVEAALAAARAAGDAAAIARAEALLARVEEARAILANPNVDPAVGRALLAAVAPEI AALAGGSYGTHHHHHH**
SES-2	AVEDALKKAEAAGDEAAIARAEILLARVEEAREILANPNVDPAVGWALLAAVAPEI AALAGGSYGTHHHHHH**
DES-1	CLCTITKEKDVKATVVVECEHAEEAKKRLEAYFNNPNVDPNANPNVQSDVEAMA AAICPGISCTLTVTSKDGTTKTATLDQPGTHHHHHH**
DES-2	KLCTIEKEKDVKLKVTVPCEEKDKAKENLDKLFNNPNVDPNANPNVDEDVQKYA EEICPGIPCTLEITSKSGETKTFTLEQPGTHHHHHH**
DES-3	SLCTIEKESDVKIKVVVECEHKEEAEKRLEALVNNPNVDPNANPNVAEDVKEMA KAICPGIPCTVEVTTKDGTTLTYTLKEPGTHHHHHH**
DES-4	ELVEIELEKDVKIKVTVEESHKEEAEKNLLALMNNPNVDPNANPNVEEDTKEFAE KICPGISCKVEITTKDGTTKEYEIEEP GTHHHHHH**
TES-1	TPETFNADMTIYENPNVGGRKVTVKGTVTKTSPDKYTITVTRNPNVPDEKYTVT GTETKLPDGNTKITGTLTGTNPNVKIQADIIVKRTDGTHHHHHH**
TES-2	APTTFNATITISENPNVPGLVETFEGTITKTTPTTYEITVKRNPNVPDEKYTVTGTE TKNSDGTTTITGKMTGTNPNVVIDATITVKKTDGTHHHHHH**
SES-2_60mer	AVEDALKKAEAAGDEAAIARAEILLARVEEAREILANPNVDPAVGWALLAAVAPEI AALAGGSGGSGGSGGSGGSGGSGSGGGLSKDIIKLLNEQVNKEMQSSNLYMS MSSWCYTHSLDGAGLFLFDHAAEEYEHAKKLIIFLNENNVPVNLTSISAPEHNFT GLTQIFQKAYEHEQNISESINNITDHAIKSKDHATFNFLQWYVAEQHEEEVLFKDI LDKIELIGNENHGLYLADQYVKGIAKSRKS**
DES-1_60mer	SLCTIEKESDVKIKVVVECEHKEEAEKRLEALVNNPNVDPNANPNVAEDVKEMA KAICPGIPCTVEVTTKDGTTLTYTLKEPGGSGGSGGSGGSGGSGGSGSGGGM QIYEGKLTAEGLRFGIVASRANHALVDRLVEGAIDAIVRHGGREEDITLVRVCGS WEIPVAAGELARKENISAVIAIGVLCRGATPSFDYIASEVSKGLADLSLELRKPITF GVITADTLEQAIEAAGTCHGNKGWEAALCAIEMANLFKSLRGGSNGTGGSGGS NGT**
TES-1_24mer	MKESFNAKITIYENPNVNGRVEEFEGKITKVSEDTYEITVKRNPNVPDEKYTVKG KEEKLEDGNTLITGKLTGTNPNVVVNADIIVKKTDGGSGGSGGSGGSGGSGGS GSGGGLSKDIIKLLNEQVNKEMQSSNLYMSMSSWCYTHSLDGAGLFLFDHAAE EYEHAKKLIIFLNENNVPVNLTSISAPEHNFTGLTQIFQKAYEHEQNISESINNITD HAIKSKDHATFNFLQWYVAEQHEEEVLFKDILDKIELIGNENHGLYLADQYVKGIA KSRKS**
CSP_J1_60mer	KLKQPADGNPDPNANPNVDPGSGNGTGGSMQIYEGKLTAEGLRFGIVASRANH ALVDRLVEGAIDAIVRHGGREEDITLVRVCGSWEIPVAAGELARKENISAVIAIGVL CRGATPSFDYIASEVSKGLADLSLELRKPITFGVITADTLEQAIEAAGTCHGNKG WEAALCAIEMANLFKSLRGGSNGTGGSGGSNGT**
CSP_J2_60mer	KLKQPADGNPDPNANPNVDPNANPNVDPGSGNGTGGSMQIYEGKLTAEGLRF GIVASRANHALVDRLVEGAIDAIVRHGGREEDITLVRVCGSWEIPVAAGELARKE

	NISAVIAIGVLCRGATPSFDYIASEVSKGLADLSLELRKPITFGVITADTLEQAIEAA GTCHGNKGWEAALCAIEMANLFKSLRGGSNGTGGSGGSNGT**
CSP_J3_60mer	KLKQPADGNPDPNANPNVDPNANPNVDPNANPNVDPGSGNGTGGSMQIYEGK LTAEGLRFGIVASRANHALVDRLVEGAIDAIVRHGGREEDITLVRVCGSWEIPVA AGELARKENISAVIAIGVLCRGATPSFDYIASEVSKGLADLSLELRKPITFGVITAD TLEQAIEAAGTCHGNKGWEAALCAIEMANLFKSLRGGSNGTGGSGGSNGT**
